# Low-cell-number, single-tube amplification (STA) of total RNA revealed transcriptome changes from pluripotency to endothelium

**DOI:** 10.1186/s12915-017-0359-5

**Published:** 2017-03-21

**Authors:** Yi-Hsuan Lee, Ya-Wen Hsueh, Yao-Hung Peng, Kung-Chao Chang, Kuen-Jer Tsai, H. Sunny Sun, Ih-Jen Su, Po-Min Chiang

**Affiliations:** 1 Department of Pathology, National Taiwan University Hospital, Taipei, Taiwan; 2 Institute of Clinical Medicine, College of Medicine, National Cheng Kung University, No. 35, Xiaodong Rd, Tainan, 70457 Taiwan; 3 Department of Pathology, College of Medicine, National Cheng Kung University, Tainan, Taiwan; 4 Institute of Molecular Medicine, College of Medicine, National Cheng Kung University, Tainan, Taiwan; 5 Division of Infectious Diseases, National Health Research Institutes, Tainan, Taiwan

**Keywords:** Transcriptome sequencing, Low cell number, Total RNA, Pluripotent stem cells, Endothelium

## Abstract

**Background:**

In addition to messenger RNA (mRNA), noncoding RNAs (ncRNAs) are essential components in cellular machineries for translation and splicing. Besides their housekeeping functions, ncRNAs are involved in cell type-specific regulation of translation, mRNA stability, genome structure, and accessibility. To have a comprehensive understanding of the identities and functions of different cell types, a method to comprehensively quantify both mRNA and ncRNA in a sensitive manner is highly desirable.

**Methods:**

Here we tried to develop a system capable of concurrently profiling both mRNA and ncRNA by polyadenylating RNA in samples before reverse transcription. The sensitivity of the system was maximized by avoiding purification from cell lysis to amplified cDNA and by optimizing the buffer conditions. The single-tube amplification (STA) system was applied to single to 100 cells of 293T cells, human pluripotent stem cells (hPSCs) and their differentiated endothelial progenies to validate its quantitative power and sensitivity by qPCR and high-throughput sequencing.

**Results:**

Using microRNA (miRNA) as an example, we showed that complementary DNA (cDNA) from ncRNAs could be amplified and specifically detected from a few cells within a single tube. The sensitivity of the system was maximized by avoiding purification from cell lysis to amplified cDNA and by optimizing the buffer conditions. With 100 human embryonic stem cells (hESCs) and their differentiated endothelial cells as input for high-throughput sequencing, the single-tube amplification (STA) system revealed both well-known and other miRNAs selectively enriched in each cell type. The selective enrichment of the miRNAs was further verified by qPCR with 293FT cells and a human induced pluripotent stem cell (hiPSC) line. In addition, the detection of other non-miRNA transcripts indicated that the STA target was not limited to miRNA, but extended to other ncRNAs and mRNAs as well. Finally, the STA system was capable of detecting miRNA and mRNA expression down to single cells, albeit with some loss of sensitivity and power.

**Conclusions:**

Overall, STA offered a simple and sensitive way to concurrently quantify both mRNA and ncRNA expression in low-cell-number samples for both qPCR and high-throughput sequencing.

**Electronic supplementary material:**

The online version of this article (doi:10.1186/s12915-017-0359-5) contains supplementary material, which is available to authorized users.

## Background

It is highly desirable to profile the expression of RNAs in a sensitive manner. Examples include sorted cells from biopsies or embryoid bodies, which can only provide a small number of cells. Even with cells from cell lines or expandable primary cultures, individual cells in cultures are known to have cell-to-cell variations, i.e., variations in cell-cycle [1] or differentiation [2] statuses. In addition, the stochastic expression of genes could be of biological importance [3]. Thus, ultralow-input or single-cell profiling will unveil valuable information.

Besides the well-known role of messenger RNA (mRNA) in determining cellular functions and fates, microRNA (miRNA), the noncoding RNA of an approximately 22-nucleotide-long sequence, binds mRNAs to regulate the level or translation of their targets [4]. miRNAs are involved in cell-cycle control [5], in cell-fate maintenance and decisions [6], and in multiple physiological/pathological processes as well, such as immunity [7]. The understanding of their roles begins with the identification and quantification of their expression. It would be even better if the quantitative information of mRNA and miRNA could be collected at the same time to characterize their interactions.

Although it is possible to profile mRNA [8] or miRNA [9] from individual cells, current options to profile both types of RNA concurrently by high-throughput sequencing are limited by the different ways of attaching adapters for reverse transcription (RT) and complementary DNA (cDNA) amplification. The 5’ capping of mRNA is incompatible with the attachment of the 5’ adapter by ligation [10], a procedure commonly used for miRNA amplification. Although it is possible to ligate a poly(A) adapter to the 3’ end of miRNA and use a template-switch (TS) reaction [11] to add the 5’ adapter to both mRNA and miRNA, the efficiency of TS is low and the use of the MnCl_2_ enhancer [12] is detrimental to the fidelity of the downstream PCR amplification.

Here, we tried to address the limitations referenced above by developing a single-tube amplification (STA) system to profile the expression of miRNAs, other noncoding RNAs (ncRNAs), and mRNA from 100 or fewer cells for high-throughput sequencing. Based on human embryonic stem cell (hESC) lines and their differentiated endothelial cells, STA revealed miRNAs to be differentially enriched in each cell type. The differential expression was further validated by conducting a quantitative polymerase chain reaction (qPCR) with an independent human induced pluripotent stem cell (hiPSC) line. With hESCs and 293T cells, we further demonstrated that STA-derived sequencing data correlated well with the data based on conventional methods in the literature regarding miRNA and mRNA expressions. Finally, STA was able to profile miRNA expression in as few as 10 cells or even in single cells at some cost of sensitivity. Overall, STA was shown to be a simple, efficient, and sensitive system to profile both polyadenylated and nonpolyadenylated transcriptomes in small numbers of cells for both high-throughput sequencing and qPCR.

## Results

### Polyadenylation in an RT buffer and high dNTP/Mg^2+^ during the TS reaction enabled efficient attachment of both adapters without a buffer exchange

**Fig. 1:**
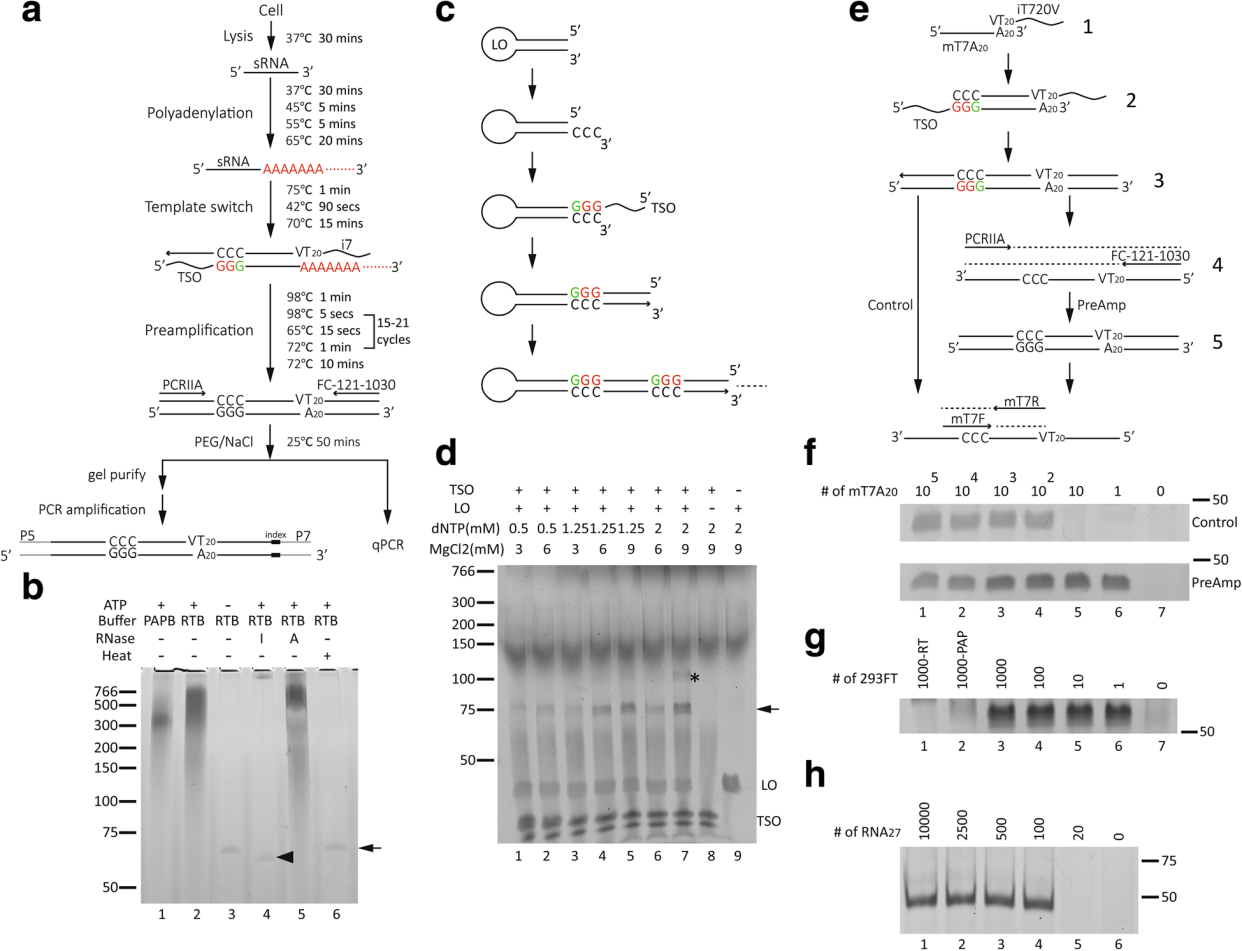
Optimizing the conditions for single-tube amplification (*STA*) of RNA. **a** Schematic representation of STA procedure. *TSO*: template-switch oligo and primer binding site for PCRIIA; *i7*: primer binding site for FC-121-1030; *P5* and *P7*: flow cell binding sites for Illumina sequencing; *red*: ribonucleic acid bases; *green*: locked nucleic acid bases. **b** Denaturing PAGE (6%) of LOrA5 under various conditions. One pmol of LOrA5 was polyadenylated with 2.5 units of PAP in 10 μl of reaction medium at 37 °C for 30 min followed by incubating at 65 °C for 20 min with the following variations: *I*: RNase If 25 units at 37 °C for 30 min after the 65 °C step; *A*: RNase A 5 μg at 37 °C for 30 min after the 65 °C step; *heat*: the reaction was heated at 65 °C for 20 min before adding LOrA5 for polyadenylation. *PAPB*: PAP buffer; *RTB*: RT buffer; *ATP*: 2.5 mM final of ATP. *Arrow*: LOrA5; *arrowhead*: LOrA5 with trimmed rA tails by RNase If. **c** Schematic representation of extending a looped DNA oligo (*LO*) with a TSO. **d** Denaturing PAGE (12%) demonstrating the effects of Mg^2+^ and dNTP concentrations on TS activity. One pmol of LO was incubated in 1 M betaine, 1 μM TSO, 1X RT buffer, 4 mM extra of dithiothreitol, 50 units of superscript II in 10 μl of reaction medium at 42 °C for 90 min followed by incubating at 70 °C for 15 min with different final concentrations of dNTP and MgCl_2_. Lanes 8 and 9 served as no-LO and no-TSO controls, respectively. *Arrow*: LO with first extended TSO; *asterisk*: LO with second extended TSO. **e** Schematic representation of detecting a DNA oligo with a 20-mer A tail (mT7A20) without (*Control*) or with (*PreAmp*) preamplification. **f** Denaturing PAGE (12%) detecting decreasing numbers of mT7A20 using STA. Various numbers of mT7A20 were subject to TS reaction as described in the Methods section. A half (5 μl) of the TS reaction was directly purified with polyethylene glycol (PEG)/NaCl, and all eluents underwent 50 cycles of PCR (*Control*, product 3 in Fig. 1e). The other half (5 μl) was preamplified with 20 cycles of PCR before purification (*PreAmp*, product 5 in Fig. 1e), and one-hundredth of the eluents was amplified with 40 cycles of PCR. **g** Denaturing PAGE (12%) of miR-19b PCR products with decreasing cell inputs. Total RNA from 1000- to 1-cell lysates of 293FT was amplified based on the STA procedure (21 cycles). One-hundredth of the purified eluents was subject to 40 cycles of PCR. *293FT-RT*: control (1000 cells) without reverse transcriptase; *293FT-PAP*: control (1000 cells) without PAP; *0*: no-cell control. **h** Nondenaturing PAGE (15%) demonstrating detection limits of STA with spike-in of small-RNA oligos. Various numbers of 27-mer RNA (RNA_27_) oligos were spiked into 100-cell lysates of TW1 hESC line. One-hundredth of the purified eluents from the 20-cycle STA procedure was subject to 40 cycles of PCR

Our goal was to maximize the sensitivity of detecting RNAs by lysing cells, attaching both adapters, and amplifying cDNA in a single tube without any purification or buffer exchange in between (Fig. 1a). After cell lysis, polyadenylation of the ncRNAs by poly(A) polymerase (PAP) served for the attachment of a 3’ adapter during reverse transcription. However, the conventional PAP buffer contained high concentrations of NaCl (250 mM) and MgCl_2_ (10 mM), which was suboptimal for the following RT step. Thus, an RT buffer containing 75 mM KCl and 3 mM MgCl_2_ was tested as a substitute for the PAP buffer for polyadenylating an oligo with a 5-ribose A tail (LOrA5). In contrast to the control without ATP (Fig. 1b, lane 3), the use of either a PAP or an RT buffer resulted in the disappearance of the unextended LOrA5 (Fig. 1b, arrow) accompanied with extensive shift-up smearing (Fig. 1b, lanes 1, 2). The successful polyadenylation of the LOrA5 was verified by the loss of shifting in the presence of ribonuclease (RNase) If, which degrades all types of RNA dinucleotides (Fig. 1b, lane 4), but not ribonuclease A, which only cuts before pyrimidines (Fig. 1b, lane 5). In addition, PAP could be inactivated efficiently at 65 °C for 20 min in the RT buffer (Fig. 1b, lane 6). Thus, the PAP was active and could be effectively heat-inactivated in the RT buffer, allowing for polyadenylation and single-buffer continuation into the subsequent RT step.

The attachment of both 5’ and 3’ adapters was based on the TS system for the purpose of profiling single-cell mRNAs [8]. To maximize the TS efficiency, a looped DNA oligo (LO) was used as a blunt-end mimic to assay for the addition of a 5’ template-switch oligo (TSO) adapter (Fig. 1c). Since nontemplate tailing of deoxyribonucleotides (dNTP) was the basis of the TS reaction, the concentration of the dNTP was increased to boost the extension. Under the standard conditions for cDNA synthesis, there was only limited extension of the looped oligo (Fig. 1d, lane 1, arrow). Increasing dNTP substrates alone, however, failed to enhance TS activity significantly (lane 3 vs 1). However, the enhancement with higher dNTP could be observed with accompanying increases in Mg^2+^ (Fig. 1d, lane 4 vs lanes 1–3), which was chelated by dNTP. The maximal efficiency was observed at 2 mM dNTP plus 9 mM Mg^2+^ (Fig. 1d, lane 7 vs all others), under which the extension of a second adapter could be observed (Fig. 1d, lane 7, asterisk). Thus, a high concentration of 2 mM dNTP plus 9 mM Mg^2+^ was chosen for the TS reaction.

### Preamplification before purification enhanced the detection sensitivity

Purification of the cDNA products from the TS reaction was required to remove oligos that would confound either subsequent qPCR reaction or library preparation. To assess the effects of cDNA purification on the sensitivity of the samples, decreasing numbers of poly(dA)-tailed oligos (mT7A20) were subjected to TS reactions and detected with a primer pair specific for the adapter-attached oligo (Fig. 1e). If the cDNA was purified directly after the TS reaction (Fig. 1e, Control route), the PCR products of correct size disappeared when the input was below 100 copies per reaction (Fig. 1f, Control) with the accompanying presence of unspecific bands (Additional file 1: Figure S1A, control, asterisks). If preamplification [8] was performed before purification (Fig. 1e, PreAmp route), the system was able to detect a few copies of mT7A20 in the reaction (Fig. 1f, PreAmp) without any unspecific products (Additional file 1: Figure S1A, PreAmp) in a quantitative manner (Additional file 1: Figure S1B). Although the sensitivity limit of a single copy was difficult to claim due to the potential inaccuracy of either the dilution or the starting amount, direct purification clearly led to an inevitable reduction in sensitivity. Preamplification before purification improved the sensitivity by ~100-fold, and the system was able to detect a few copies of the starting material. The high sensitivity of the TS reaction plus preamplification was further supported by the presence of amplified cDNA smears with serial dilutions of 293FT lysates in denaturing polyacrylamide gel electrophoresis (PAGE) (Additional file 1: Figure S1C). Even though the cDNA smear was faint (Additional file 1: Figure S1C, lane 6 vs 7), miR-19b was detectable in the lysate equivalent to single cells only in the presence of both RT and PAP (Additional file 1: Figure S1D, lane 6 vs 1, 2, and 7). Taken together, it was possible to perform cell lysis, polyadenylation, a TS reaction, and preamplification in a single tube, and the single-tube amplification (STA) was able to detect noncoding targets in individual cells. To evaluate the absolute number of short RNAs that could be detected by STA, decreasing numbers of RNA oligos were spiked into 100 TW1 hESCs for STA with 20 cycles of preamplification. The cDNA of the spiked-in RNA oligo could be detected quantitatively (Additional file 1: Figure S1E, *R* ^2^ = 0.96) when the input number was 100 or higher (Fig. 1h, 10,000, 2500, 500, and 100).

### STA was compatible with high-throughput sequencing to quantify human pluripotent stem cell (hPSC)-enriched miRNAs from 100 cells

**Fig. 2:**
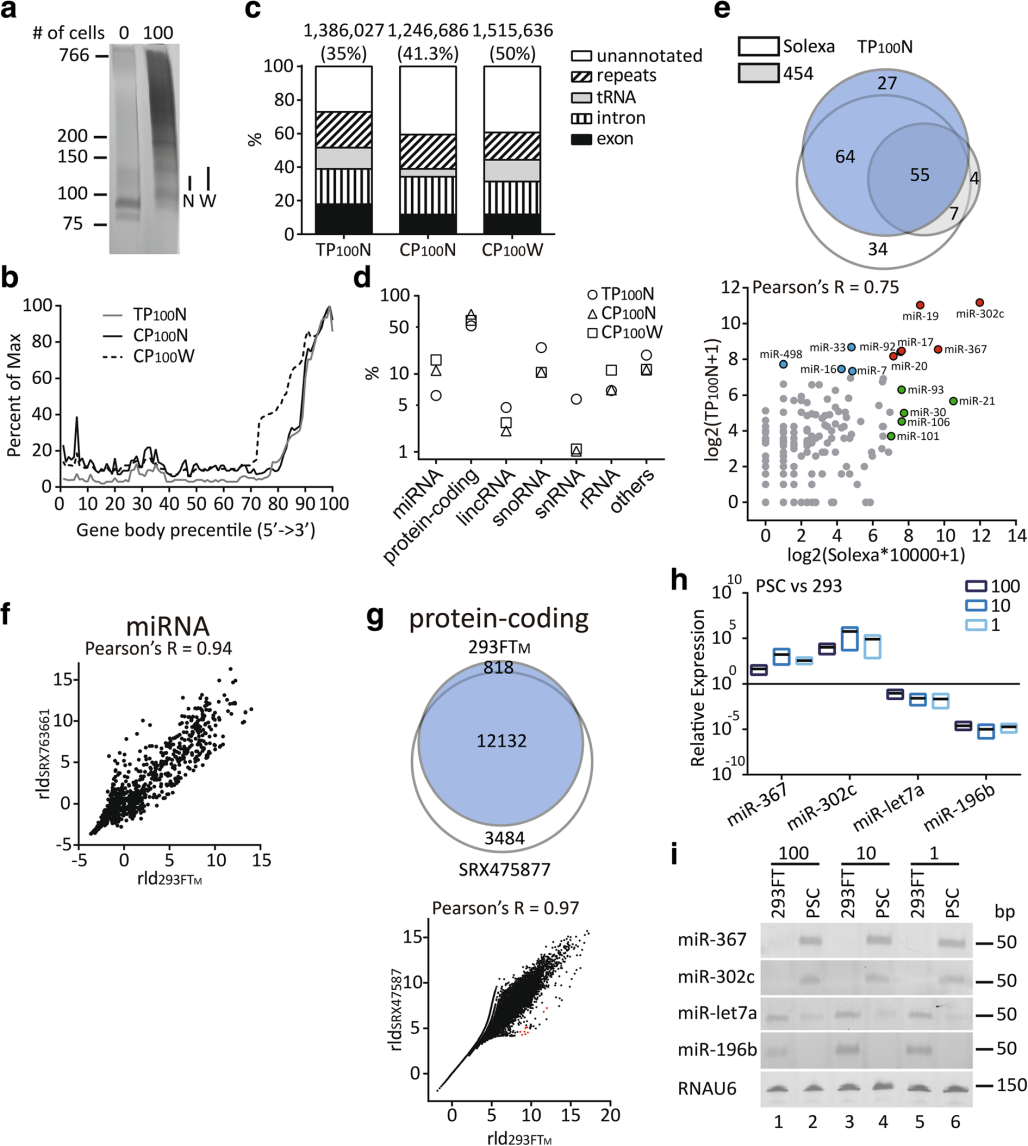
High-throughput profiling of hPSC and 293-cell transcriptomes based on STA. **a** Representative denaturing PAGE (6%) of the preamplified cDNA from 0 (*left*) and 100 (*right*) hESCs. One hundred hESCs were sorted directly into 2 μl of lysis buffer and subject to STA. All 15-cycle preamplified products were purified with PEG/NaCl and electrophoresed. Two different widths (*N*: narrow; *W*: wide) of gel slices were cut for library preparation. The 21-cycle preamplified and purified products of lysis buffer alone served as a no-cell control. **b** Gene body coverage chart of all aligned reads against all transcripts based on GENCODE v22. **c** The distribution of reads among genomic features. Strandedness was taken into account for counting in the order of exon (exon, GENCODE v22), intron (transcript minus exon, GENCODE v22), tRNA (evidence-based annotation of tRNA, GENCODE v22), and repeat (GRCh38-based RepeatMasker track on UCSC Genome Browser) features, and the rest was considered to be unannotated. The numbers on top of each column indicate reads successfully aligned to the GRCh38 genome assembly. The percentages in parentheses are the numbers of successfully aligned reads divided by the total reads in the respective libraries. **d** The RNA-type summary of the exon reads in the three libraries based on GENCODE v22. The percentages indicate the numbers of reads divided by the number of exon reads. **e** *Upper*: Venn diagram showing the overlap of the miRNAs identified (values >0) in TP_100_N and two other hESC reference sequencing data sources (Solexa and 454) in the literature. *Lower*: Scatter plot demonstrating the association of miRNA quantifications between TP_100_N and the referenced Solexa data. Each value of the reference sample was multiplied by 10,000 because only normalized data were available with the reference samples. *Blue*, *green*, and *red* colors indicate the top 10 most highly expressed miRNAs in TP_100_N, reference sample, and both, respectively. **f** Scatter plot demonstrating the association of rlog-normalized (*rld*) miRNA quantifications of 293 cells between a library prepared with STA (*293FT*_M_) and that with conventional ligation-based method (*SRX763661*). Identical genome alignment (GRCh38) and feature assignment (GENCODE v22) were performed, and all miRNAs (raw counts >0) were used for rlog transformation (blind = TRUE) with DESeq2. **g** *Upper*: Venn diagram showing the overlap of protein-coding RNAs identified (values >0 in each sample) in 293FT_M_ and those in reference RNA-Seq data (SRX475877) from 293 cells. *Lower*: Scatter plot demonstrating the association of rlog-normalized (rld) protein coding-RNA quantifications of 293 cells between a library prepared with STA (293FT_M_) and reference SRX475877 RNA-Seq data. Identical genome alignment (GRCh38) and feature assignment (GENCODE v22) were performed, and all protein-coding RNAs (raw counts >0) were used for rlog transformation (blind = TRUE) with DESeq2. Representative histone-coding RNAs enriched in the STA-based library are highlighted in *red*. qPCR (**h**) and denaturing PAGE (12%) of the semi-quantitative PCR products (**i**) of miR-302c, miR-367, miR-let7a, and miR-196b. hiPSC (DF19-9-7T) and 293FT cells were lysed, and 100-, 10-, and 1-cell equivalents of lysates were used for STA. Preamplification of 15, 18, and 21 cycles were used for 100-, 10-, and 1-cell lysates, respectively, and 1/500 of the preamplified and purified eluents was used for qPCR. The optimal cycle numbers (threshold cycle plus 4) for semi-quantitative PCR in **i** were based on the qPCR analysis in **h**. RNAU6 served as a loading control

To show if STA was able to comprehensively profile miRNA expression in a few cells, two different lines of hESCs, TW1 and Ch8, were sorted directly into a lysis buffer for cDNA amplification. Successful amplification was identified by the smearing in denaturing PAGE compared with the no-cell control (Fig. 2a, 100 vs 0). Two different band widths were collected (Fig. 2a, N and W) for library preparation and sequencing to see if the width affected the sequencing output. Most of the sequencing reads were mapped to the 3’ transcriptome region (Fig. 2b), indicating that the transcripts remained intact during polyadenylation. For the 35.0–49.9% of reads mapped to a genome, however, tRNA (4.7–13.0%), repeats (16.4–21.4%), and intronic reads (19.7–22.6%) accounted for the majority of the mapped reads (Fig. 2c and Additional file 2: Table S1). Among the abundant unannotated reads (27.0–40.6%), most of them (~90%, Additional file 3: Table S2) were rRNA transcripts not annotated in GENCODE v22 (Additional file 4: Figure S2A3). Other unannotated reads were located on transcripts in Expressed Sequence Tag (EST) or human mRNA databases (Additional file 4: Figure S2A1, 2, 4), near the start of tRNA (Additional file 4: Figure S2A5) or belonged to antisense sequences on the exons or introns of transcripts (Additional file 4: Figure S2A6). Of the 11.6–17.8% of the mapped reads that belonged to exon reads, around 70% of them were represented by protein-coding genes (41.1–58.1%), small nucleolar RNAs (snoRNAs, 10.5–21.6%), and miRNAs (5.3–15.1%) (Fig. 2d and Additional file 2: Table S1). The low miRNA read percentages (1.7–3.0%) among the mapped reads compared with those (2–62%, medium 19%) derived using conventional procedures from a large amount of samples [13] could be explained by performing size selection after attaching both 5’ and 3’ adapters with STA. The combined length of ~105 bp made the libraries containing miRNAs difficult to isolate from those containing RNAs of similar sizes (snoRNAs or small nuclear RNAs (snRNAs)). In addition, performing size selection after STA increased the carryover of protein-coding mRNAs due to their fragmentation during RT and consequent co-amplification and co-purification, which was exemplified by the prominent 3’ GAPDH peaks (Additional file 4: Figure S2A7).

The miRNA quantifications based on STA showed strong correlations between the expressions of the two hESC lines (Additional file 4: Figure S2B, upper, TP_100_N vs CP_100_N, Pearson’s *R* = 0.98). As a comparison, the miRNA sequencing data from sorted hESCs based on conventional library preparation and Solexa and 454 sequencing systems in the literature [14] were employed. The miRNAs identified using STA showed a significant overlap with those identified using the conventional method (Fig. 2e, Additional file 4: Figure S2C, D, upper panels). The Solexa data were further used to acquire correlation coefficients against STA as a result of the higher number of miRNAs detected relative to those detected using 454. Moderate to strong correlations could be observed between the reference data and the STA data (Fig. 2e, Additional file 4: Figure S2B–D, lower panels, Pearson’s *R* = 0.68–76 vs 0.94 of Solexa vs 454). Further, significant co-occurrence of top expressers could be demonstrated irrespective of sample sources and methods (Fig. 2e, Additional file 4: Figure S2B–D, red dots in scatter plots).

To further validate STA, amplified 100-cell cDNA from a common cell line, 293FT, was run against mock libraries prepared from 21- and 27-mer RNA oligos (Additional file 4: Figure S2E, lanes 1–4) to refine the size selection (Additional file 4: Figure S2E, lane 7, M). The percentages of miRNA (5.2%), tRNA (16.0%), and snoRNA (18.0%) were not much different from those of hESCs (Additional file 2: Table S1, 293FT_M_ vs PSC), and protein-coding RNA still accounted for the largest proportion of exon reads (31.9%). The expression of miRNA (293FT_M_) based on STA showed a decent match with that (HEK293) based on conventional ligation-based methods in terms of genes identified (Additional file 4: Figure S2F) and quantification (Fig. 2f, 293FT_M_ vs SRX763661 [15], Pearson’s *R* = 0.94; Additional file 4: Figure S2G, left, 293FT_M_ vs SRX556516 [16], Pearson’s *R* = 0.91). However, when both sequencing data were based on STA, a higher correlation was observed even if a 293 T cell line from a different source was used (Additional file 4: Figure S2G, right, 293FT_M_ vs 293T_M,_ Pearson’s *R* = 0.98). Although the identical culture conditions of 293FT and 293T could account for the higher correlation of STA-based data, preferential detection of individual miRNAs with STA- and ligation-based methods could equally lead to the phenomenon.

Besides miRNA, long intergenic noncoding RNA (lincRNA) expression based on STA also showed good correlation with results based on ligation-based methods (S2H, 293FT_M_ vs SRX763661 and SRX556516, Pearson’s *R* > 0.95). Further, quantitation of protein-coding RNA, the most abundant type of exon reads, in STA-based sequencing data also showed good correlation with that in the mRNA-Seq data from purified poly(A) + mRNA [15] (Fig. 2g, Venn diagram and scatter plot, Pearson’s *R* = 0.97). The enrichment of histone genes (Fig. 2g, lower, red dots) in the STA-based output demonstrated the advantage of STA in detecting nonpolyadenylated protein-coding transcripts [17]. Finally, by comparing the differential expression of protein-coding transcriptomes of hESCs and 293FT/T, *NANOG*, *SOX2*, and *POU5F1*, genes well known to be expressed in hESCs, could be revealed (Additional file 4: Figure S2I, left).

By comparing the differential expression of miRNA between hESCs and 293FT/T cells (Additional file 4: Figure S2I, right), two miRNAs, miR-302c and miR-367, were picked to validate their enrichment in an hiPSC line, DF-19-9-7T, by qPCR. Compared with the control 293FT cells, both miRNAs demonstrated significant enrichment (Fig. 2h, miR-302c and miR-367). Conversely, miRNAs known [18, 19] to be abundant in 293FT/T cells (Additional file 4: Figure S2I, right) showed reversed enrichment (Fig. 2h, miR-let7a and miR-196b). Further, the differential expression could be observed down to the lysate from single-cell equivalents (Fig. 2h, 10 cells and 1 cell). Finally, the PCR products of miRNAs and the reference RNAU6 were located at predicted sizes through the denaturing PAGE (Fig. 2i).

### The RNA expression profiles using STA clearly blindly segregated ESCs and endothelial cells

**Fig. 3:**
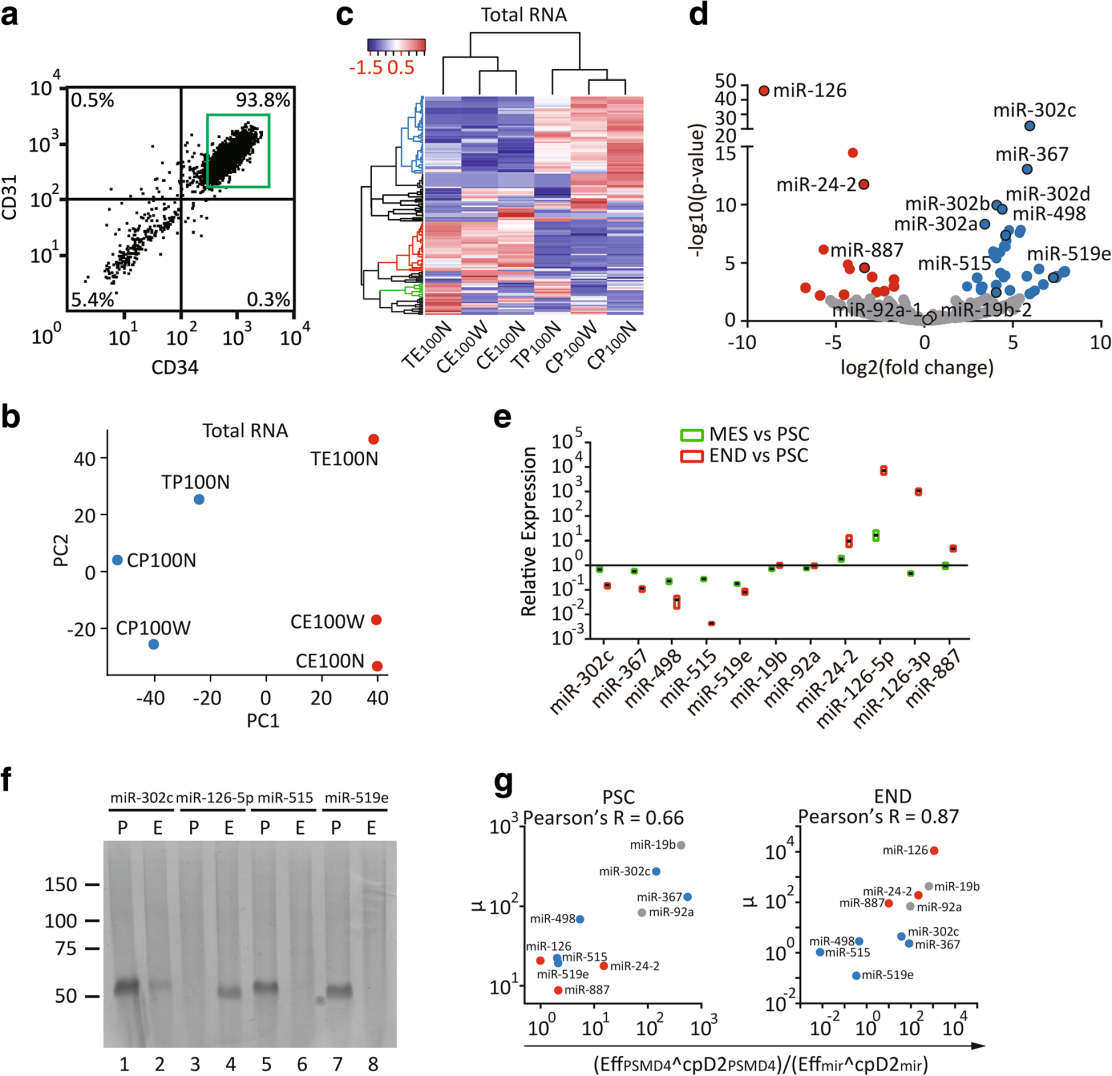
Profiling the differential expression of RNA between hPSCs and the differentiated endothelial cells using STA. **a** Fluorescence-activated cell sorting for CD31+/CD34+ cells. hESCs (20,000) were induced for 2 days into mesoderm. The induced mesodermal cells (20,000) were replated into a vascular mixture for another 3 days before sorting. The cells in the *boxed area* were seeded directly into 2 μl of lysis buffer for STA. **b** Unsupervised principal component analysis (*PCA*) based on the expression of total RNA of 100 hESCs and the differentiated endothelial cells. Genes (GENCODE v22) with summed counts >20 across six samples were rlog-transformed with DESeq2 (blind = TRUE), and the output was subject to PCA with default parameters. **c** Unsupervised heat map of the data in **b**. The top 150 variable genes of the rlog-transformed counts in **b** served as the input for heatmap3 analysis with default parameters. *Blue*, *red*, and *green* clusters indicate genes enriched in hESCs, endothelial cells, and the TW1 cell line, respectively. **d** Volcano plot showing the differential expression of miRNA between 100 hPSCs and endothelial cells. miRNA (GENCODE v22) with summed counts >1 across six samples were included for differential-expression analysis with DESeq2. *Colored dots* indicate log2 fold change >1 and an adjusted *p* value <0.05. miRNAs for further validation and in miR-302/367 cluster are highlighted with a *black border*. qPCR (**e**) and representative denaturing PAGE (12%) of the amplified products (**f**) of miR-302c, miR-367, miR-498, miR-515, miR-519e, miR-19b, miR-92a, miR-24-2, miR-126-3p, miR-126-5p, and miR-887. DF19-9-7T cells and their day-2 differentiated mesodermal (*MES*) and day-5 endothelial (*END*) progenies were lysed for STA (15 cycles) and qPCR (**e**) or semi-quantitative PCR (**f**). The differentiation procedure was performed as in **a**, and 100 cells at the mesoderm and endothelial stages were harvested directly for STA (15-cycle preamplification). One-hundredth of the preamplified and purified eluents was used for qPCR and PCR. The optimal cycle numbers (threshold cycle plus 4) for semi-quantitative PCR in **f** were based on the qPCR analysis in **e**. One hundred undifferentiated DF19-9-7T cells served as the reference sample. PSMD4 served as a loading control for analyses with qpcR. **g** Scatter plots correlating the expressions of the RNA-Seq data with the qPCR quantifications. *y*-axis represents the regressed means of the hESC data by DESeq2 in **d**. *x*-axis (μ) represents the relative expression of the respective miRNAs normalized by the PSMD4 of the DF19-9-7T cell line in **e**. *Eff*: efficiency; *cpD2*: threshold cycle; *left panel*: hPSC; *right panel*: differentiated endothelial cells

To demonstrate the power of STA to comprehensively compare the transcriptomes between different cell types, sequencing data from the hESCs and their differentiated CD31+/CD34+ endothelial progenies (Fig. 3a) were compared (see Additional file 5: Figure S3A and Additional file 2: Table S1 for summaries of the endothelial datasets). Based on the expression of total RNA in an unsupervised manner, the endothelial samples segregated well from the hESCs according to principal component (PC) 1 and cluster dendrogram analyses (Fig. 3b, c, P vs E). Similar segregation of the two cell types was evident by the expression of miRNA alone (Additional file 5: Figure S3B, C). By the expression of total RNA, the two hPSC lines could be separated from each other using PC2 (Fig. 3b, T vs C). Consistent with the segregation, the TW1 cell line showed an extra group of enriched genes relative to Ch8 (Fig. 3c, green cluster, T vs C) in addition to hESC- and endothelium-enriched genes (Fig. 3C and Additional file 5: Figure S3C, the blue and red clusters, respectively).

### STA revealed miRNAs specifically enriched in PSCs and their differentiated endothelial cells

Differential expression of the sequencing data revealed that the miRNAs expressed selectively in either the hESCs or endothelial cells (Fig. 3d, the blue and red dots, respectively). Some of the enriched miRNAs have already been documented. For example, the miR-302/367 cluster (Fig. 3d and Additional file 5: Figure S3D, panel 3) is known to be abundant in hESCs in maintaining pluripotency [14]. In addition, miR-126, a gene regulating vascular leakiness [20], was found to be the most abundant (GSE93672_gene_counts.csv) miRNA in endothelial samples (Fig. 3d and Additional file 5: Figure S3D, miR-126). In addition to the known miRNAs, the STA revealed other differentially enriched miRNAs, such as miR-887/miR-24-2 in endothelial cells and miR-519e in hPSCs (Fig. 3d and Additional file 5: Figure S3D). The selective enrichment of miRNA by sequencing was further validated by qPCR on the DF19-9-7T hiPSC line and its endothelial progenies (Fig. 3e, red bars, where miR-19b and miR-92a served as housekeeping controls; see Fig. 3f and Additional file 5: Figure S3E for the miRNA products obtained through semi-quantitative PCR in a denaturing PAGE). At the mesodermal stage, the intermediate between hPSCs and endothelial cells, the levels of the miRNAs tended to remain in between (Fig. 3e, green bars). Finally, the moderate to strong correlation between the quantifications by sequencing and qPCR of different hPSC lines supported that the STA was technically solid and consistent (Fig. 3g).

### At the expense of sensitivity, the STA provided quantitative transcriptome information from 10 cells and from a single cell

**Fig. 4:**
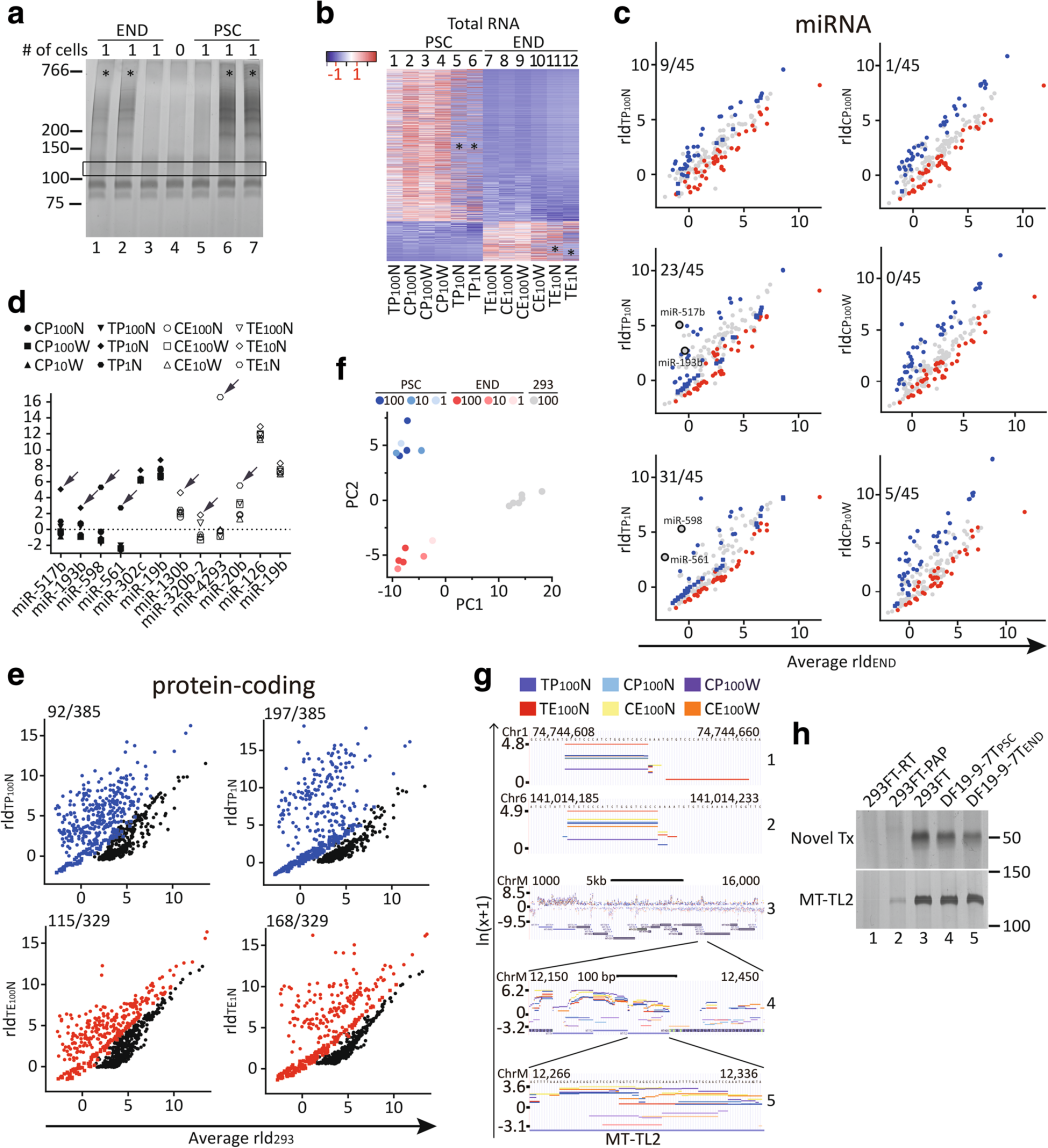
Probing the detection limit with 10 to single cells. **a** Representative denaturing PAGE (6%) of the PEG/NaCl-purified, amplified cDNA libraries from single hPSCs or sorted endothelial progeny. Differentiation, sorting, and STA were performed as in Fig. 3a. For single cells, only cDNAs successfully amplified after 21 cycles of preamplification (*asterisks*) were size-selected (*rectangular box*) for library construction. **b** The supervised heat map of total RNA expression of all 12 PSC and END samples. Genes (summed count across all 12 samples >20) from all 12 samples were used for differential-expression analysis with DESeq2. The rlog-transformed counts with lowest *p* values (1000) were ranked by log2 fold changes and served as input for heatmap3 without rearranging column and row dendrograms. **c** Scatter plots of rld_miRNA_ from individual samples of hESCs against the averaged rld_miRNA_ of six endothelial samples. Only miRNAs with summed counts >20 across 12 PSC and END samples were included for DESeq2 analysis. *Colored dots* represent miRNAs enriched in hPSCs (*blue*) or endothelial cells (*red*) as defined in Fig. 3d. The *blue dots* were transformed into *squares* when the values of (rld – average rld)/log10 (10 + average rld) were less than or equal to 0.4. The ratio of blue squares over total blue (*square* + dots) is indicated in the *upper left corner*. Exemplary aberrant expressers in low-input samples are highlighted with *black borders*. **d** rld of aberrantly detected miRNAs (highlighted dots in **c**) in individual low-input (*arrows*) vs those in the other samples of hESCs (P) and endothelial cells (E). **e** Scatter plots of rld_protein-coding_ from individual samples of hESCs (*upper*) and endothelial cells (*lower*) against the averaged rld_protein-coding_ of the six 293T samples. *Colored dots* represent protein-coding genes differentially expressed (*p* < 0.01) in hESCs (*blue*), endothelial cells (*red*), and 293T cells (*black*) by DESeq2 analysis. Protein-coding genes with summed counts >20 across the 12 samples (the 6 hESC and the 6 293T samples, or the 6 endothelial and the 6 293T samples) were included for differential-expression and rlog-transformation analyses. The *blue* and *red dots* were transformed into *squares* when the count of a particular gene was 0. The ratios of squares over (squares + dots) are indicated in the *upper left corner*. **f** Unsupervised PCA based on the mature miRNA counts of all 18 samples. miRNAs (mature miRNA by accession number, miRBase v21) with summed counts >500 across the 18 samples were rlog-transformed with DESeq2 (blind = TRUE). The transformed rld served as the input for PCA with default parameters. **g** Visualization of novel (panels 1, 2) and mitochondrial (panels 3–5) transcripts in the UCSC Genome Browser. Each curve represents reads per million (*RPM*)-normalized wiggle output of the libraries against the GRCh38 genome assembly. **h** Validation of the transcripts by denaturing PAGE (12%) for the novel (*upper*) and mitochondrial (*lower*) transcripts. One-hundredth of the preamplified cDNAs in Figs. 1g and 3f was used for 30 cycles of PCR

To probe the sensitivity limit of the STA, both 10 and single hESCs or differentiated endothelial cells were sorted and subjected to STA and sequencing (see Additional file 6: Figure S4A and Additional file 2: Table S1 for the sequencing summaries). Although amplified cDNA as smears could be consistently identified from the 10-cell input, amplified products were detected in ~50% of the wells seeded with single cells (Fig. 4a, asterisks). The 50% chance of obtaining libraries was likely due to sorting, because the detection of amplified cDNA was mostly all or none in individual wells after 21 cycles of preamplification. Generally, the gene expression trends from low-input libraries matched those from 100 cells considering both total RNA (Fig. 4b, 10 or 1 vs 100) and miRNA (Additional file 6: Figure S4B, 10 or 1 vs 100). However, reduction of sensitivity was identified in the low-input samples (Fig. 4b and Additional file 6: Figure S4B, asterisks). By comparing the miRNA expression of the individual samples against the average of the other cell types, the low-input samples tended to miss enriched genes of their type (Fig. 4c and Additional file 6: Figure S4D, mean ± SEM of the percentages of squares over colored dots, 100 vs 10 vs 1, 7.5 ± 3.1% vs 30.9 ± 8.5% vs 62.9.0 ± 6.0%). In addition, aberrant over representation of miRNAs was identified in the low-input samples (Fig. 4c and Additional file 6: Figure S4D, encircled and labeled). The aberrant detection of these miRNAs could be further demonstrated by plotting against the quantities found in other samples (Fig. 4d, arrows indicating aberrant detections). Nevertheless, using the principal components derived from the 100-cell inputs as a predictor (Fig. 3b), the low-input samples from the PSCs and endothelial cells could still be segregated by PC1 (Additional file 6: Figure S4C, P vs E), albeit with shorter distances apart for some samples (Additional file 6: Figure S4C, TP_10_N and TP_1_N vs E). Regarding individual genes, the expression of abundant miRNA was detected in single cells (Additional file 6: Figure S4E, miR-126, miR-887, miR-302c, miR-515-1, 1 cell). However, for some less abundant miRNAs, loss of peaks was identified in some samples with either 1 cell or 10 cells (Additional file 6: Figure S4E, miR-24-2 and miR-519e). Regarding protein-coding genes, the loss of detection was also observed with 1-cell samples, as evidenced by increased zero-count genes that were enriched in particular cell types (Fig. 4e, TP/E_100_N vs TP/E_1_N). In addition, the wider dispersion of the detected protein-coding genes in the single-cell samples indicated reduced quantitative power with single-cell samples (Fig. 4e, TP/E_100_N vs TP/E_1_N). Nevertheless, even with these issues, the expression profiles of mature miRNA in 10 or single cells co-segregated with those of 100 cells in a cell-type-dependent manner by unsupervised PCA (Fig. 4f, PSC vs END vs 293). The observation suggested that STA would be useful in sorting and comparing single-cell transcriptomes in a mixed and heterogeneous cell population.

Although the sequencing data from wider size-selection pieces tended to cover a broader region in the 3’ transcript regions (Fig. 2b, Additional files 5 and 6: Figures S3A and Figure S4A), we could not identify significant differences in the sequencing outputs regarding the percentages of aligned, exon, or miRNA reads (Additional file 2: Table S1). In addition, the miRNA expression levels between the outputs from two widths were still highly correlated (Additional file 6: Figure S4F). However, if the size selection was further refined by running libraries for longer times in higher percentage PAGE gels (12%) and by dividing into 5 different size ranges (Additional file 4: Figure S2E, H, M, L, 21, 27), the 293FT libraries prepared from higher size ranges contained lower percentages of miRNA (Additional file 2: Table S1, miRNA, 2.5–2.9% vs 5.2–6.9%, 293FT_H/27_ vs 293FT_M/L/21_) and higher percentages of tRNA (Additional file 2: Table S1, tRNA, 21.6–51.9% vs 7.7–16.0%, 293FT_H/27_ vs 293FT_M/L/21_). In addition, the library from the highest positions (293FT_H_) also contained higher proportions of lincRNA (6.7%) and antisense RNA (7.8%) compared with the other libraries (Additional file 2: Table S1, 293FT_H_ vs the others). The correlation of miRNA expression between 293FT_H_ and 293FT_M_ was not as good as those between the other ranges and 293FT_M_ (Additional file 6: Figure S4G, left upper vs the other three, Pearson’s *R* = 0.95 vs 0.98–0.99). Although the library from the lowest position (293FT_L_) and 293FT_M_ had similar percentages of miRNA among exon reads (Additional file 2: Table S1, miRNA, 293FT_L_ vs 293FT_M_) and correlated well in miRNA quantification (Additional file 6: Figure S4G, lower left), the percentage of successfully aligned reads (1,897,606/28,442,806 = 6.7%) of 293FT_L_ was much lower that of 293FT_M_ (13,499,417/27,717,138 = 48.7%), possibly due to the abundant short reads that could not be aligned in 293FT_L_. The quality of the library from the 21-mer location (293FT_21_) was similar to that from 293FT_M_ regarding the percentage of successfully aligned reads, the miRNA percentage among exon reads, rRNA commination (Additional file 2: Table S1, 293FT_21_ vs 293FT_M_), and the correlation of miRNA expression (Additional file 6: Figure S4G, right lower). Overall, these facts indicated that the sequencing results with STA tolerated some variations during size selection. However, the use of mock RNA-oligo libraries of known size could improve library quality in terms of enriching desirable RNA species and reducing fail-to-align reads.

### STA was capable of detecting both novel and mitochondrial transcripts

By excluding reads that aligned to features in the GENCODE v22 and RepeatMasker on UCSC Genome Browser, STA revealed novel short transcripts and extensive mitochondrial reads (Fig. 4g, panels 1, 2 and 3–5, respectively). These transcripts could be detected in multiple cell lines and types, and the presence of an amplified product only in the presence of both RT and PAP corroborated that they were nonpolyadenylated RNA in nature (Fig. 4h, lanes 3–5 vs 1, 2).

Taken together, STA bypassed the lengthy preparation of RNA or the complicated buffer exchanges mandatory with conventional methods. The single-tube system made the system ultrasensitive in quantifying miRNAs, as well as other noncoding and organellar RNAs, down to individual cells. The validity and applicability of STA were demonstrated by qPCR with multiple independent cell types and by matching with existent databases from conventional methods.

## Discussion

Several methods have been proposed to add adapters for amplifying small RNA (sRNA): sequence-specific annealing [21], ligation [22], ligation and TS [23], polyadenylation [24], and polyadenylation and TS [25]. The use of sequence-specific annealing is limited to RNA of known sequences. Adapter ligation is known to depend on the sRNA sequence [26]. In addition, methods based on ligation usually require multiple rounds of purification steps to avoid adapter carryover, which further undermines sensitivity. Although adapter decapping bypasses the requirement of purification, the ligation-based method is still affected by the 5’-phosphorylation status of target RNA and requires a longer time and more steps to accomplish than STA does. Compared with current TS and ligation-based methods, the efficiency of the TS step of STA remained high even if the targets were 5’-nonphosphorylated (Fig. 1f and h, nonphosphorylated DNA and RNA, respectively), probably due to the high dNTP/Mg^2+^ level that increased nontemplate nucleotide addition by reverse transcriptase. The phosphorylation-independent nature of STA would be useful if the target RNA is capped like mRNA [27] or nonphosphorylated. Tailing the 3’ ends using polyadenylation ameliorated the formation of adapter dimers even with single cells as the input. Although polyadenylation also introduces biases associated with RNA sequences and structures, such as 2’-*O*-methylated RNA at the 3’ ends [28], the STA libraries show decent matches with those from conventional methods in terms of miRNA expression (Fig. 2e, f).

The single-tube system avoids buffer exchange or in-between adapter removal and minimizes sample loss. Overall, STA reduces the starting material requirement from ~10^3^ cells (5–10 ng of total RNA) to 1–100 cells. The improved sensitivity is of biological value because individual cells in the same culture have transcription variations that reflect cell cycles or differentiation statuses [2]. In addition to its high sensitivity, STA requires less than 5 h to obtain preamplified libraries for qPCR or for size selection. The simple procedure and short hands-on time also mean that multiple samples can be prepared together to minimize batch-to-batch variations. Therefore, STA, with its improved sensitivity and speed, could complement or even replace conventional ligation-based methods.

In addition to profiling miRNAs, STA also offers the possibility of profiling all RNAs, as demonstrated by the high correlation of protein coding-RNA expression between STA-based and conventional mRNA-Seq libraries, and by the abundant mitochondrial reads in our datasets. However, the profiling of entire transcriptomes without size selection is limited by the presence of abundant transcripts, such as rRNA, tRNA, and snoRNA. Although it is possible to deplete the undesirable RNAs using pull-down or nuclease treatments [29], there will be an accompanying loss of target RNAs. It would be possible to remove these sequences by annealing and pull-down or size selection after preamplification. However, both methods still suffer from losing target RNAs along with undesirable ones. The most straightforward solution would be an increase in sequencing depths. Not limited to profiling cellular transcriptomes, STA could be theoretically adapted to profile eluted RNA from RNA immunoprecipitation, circulating or secreted RNA, or prokaryotic transcriptomes.

STA depends on efficient polyadenylation of RNAs at their 3’ ends. However, RNAs might be buried in protein complexes or secondary RNA structures that block PAP access. We tried to remove protein hindrances by treating cells with proteinase K before polyadenylation. The extension of recessed or blunt ends was addressed using a stepwise increase in incubation temperature during polyadenylation. It might be possible to further expose the refractory ends with low concentrations of denaturants, such as guanidium thiocyanate, that also help the RT reaction by loosening second structures during cDNA synthesis [30]. For circular RNAs or RNAs with blocking modifications at the 3’ ends, such as Piwi-interacting RNA (piRNA) [28], limited fragmentation might be required for PAP access. For mature RNAs with blocked 3’ ends, the ability of STA to detect their precursors will still make it possible to obtain quantitative information. However, STA is unable to discern isoforms of RNA when the only difference among them is one or more A bases at their ends [31]. In this case, libraries based on ligation or STA based on poly(U) polymerase would work around the limitation.

The quality of the libraries from a single cell or from 10 cells was as not good as that from 100 cells. The loss of detection power could have been caused by deteriorated RNA quality during staining and sorting. The lengthy sorting and staining processes could be avoided by direct lysis of single cells using a limiting dilution. The successful amplification of cDNA could be identified by running part of the library with denaturing PAGE, and the identity of each library could then be verified by the expression of cell-type-specific genes. Since <100 copies of DNA could be amplified (Fig. 1f), the bottleneck for assaying diluted RNAs (<100 copies of target per reaction) could be the loss of targets during heat inactivation of PAP or RNA degradation from either endogenous or exogenous RNase. The PAP-inactivation step could be bypassed by replacing the 3’-OH with unextendable -H at the 3’ end of TSO. The problems of RNases might be addressed by including tiny amounts of bacterial rRNA or other decoy RNA that could be simply filtered during subsequent alignment or during the size-selection step.

Even if the quality of low-input sequencing data cannot be improved, the sequencing data from single cells will still be able to reflect their cell of origin (Fig. 4f). The counts from multiple single-cell libraries could be analyzed collectively based on the expression of signature genes. The digital-to-quantitative transformation makes it possible to filter out aberrant expressions (Fig. 4d) and to generate the genuine expression profiles of each cell type. Compared with the ligation-based, single-cell sequencing of sRNA developed recently [9], STA has the advantages of a simple procedure, immunity to the 5’-phosphorylation status, and the capability of detecting both noncoding and polyadenylated RNA concurrently. The concurrent detection allows for correlating surface markers with the expression of ncRNAs. The correlation allows for identifying novel cell types and for enrichment by sorting for further characterization.

As a proof of the effectiveness of the methodology, STA revealed the changes in miRNA expression during the transition from hPSCs to endothelial cells across three different hPSC lines. Some miRNAs known to be highly enriched in hPSCs, e.g., miR-302/367 [14], or endothelial cells, e.g., miR-126 [20], were clearly identified with STA. Several others could still be of biological or clinical interest. For example, miR-24 has been shown to be associated with aortic aneurysms [32] and to be essential for embryonic hematopoiesis [33]. The refinement of miR-24 expression specifically in endothelium pinpoints the central role of endothelial cells in mediating aortic inflammation. Together with the requirement of miR-24 for embryonic hematopoiesis, its specific enrichment in endothelial cells also suggests that miR-24 is critical for the identity or hemogenic potential of embryonic endothelium. Further, the identification of hPSC-enriched miR, such as the miR-498/515/519e cluster, also provides an interesting target for roles in pluripotency or differentiation.

## Conclusions

Overall, STA is a simple, sensitive, and efficient way to profile total RNA expression with the potential to complement conventional ligation-based methods. With minor modifications, STA can be applied to amplify and detect minute amounts of RNA from different sources. Its ability to detect and quantify both mRNA and ncRNA expressions currently allows for characterizing the interaction of the RNA species in a way not achievable in the past. Finally, the application value of STA was verified by providing RNAs selectively enriched in either PSCs or endothelial cells, which serve as a good resource for further investigation.

## Methods

### Materials

The materials used were poly(A) polymerase (Enzymatics); SYBR Gold, SuperScript II RT and SUPERase In RNase Inhibitor (ThermoFisher); Sera-Mag SpeedBead Carboxylate-Modified Magnetic Particles (GE Healthcare); Taq DNA polymerase (pAKTaq was a gift from David Engelke, Addgene plasmid # 25712); KAPAHiFi DNA Polymerase (Kapa Biosystems); αhCD31 and αhCD34 (BD Biosciences). The oligo sequences for STA and PCR are summarized in Additional file 7: Table S3. The reagents and method used for the differentiation and sorting of the endothelial cells from hPSCs were identical to those of Ref. [34].

### STA

The cells were either diluted into phosphate-buffered saline (PBS) with 0.1% polyvinyl alcohol (PVA) before lysis or sorted directly into a lysis buffer (0.1% Triton X-100 with 100 μg/ml of proteinase K). After incubation at 37 °C for 30 min and inactivating proteinase K by adding 0.25μl of 10mM PMSF in DMSO, the lysate was polyadenylated in a final 4 μl of 1X RT buffer (50 mM Tris-HCl, 75 mM KCl, 3 mM MgCl_2_, 10 mM dithiothreitol (DTT), pH 8.3) with 100 μM of ATP, 1 unit of SUPERase In, and 1.25 units of PAP (37 °C for 15 min, 45 °C for 5 min, 55 °C for 5 min, and 65 °C for 20 min). The TS reaction was performed by supplementing 0.5 μl of 20 μM i7T20V oligo, heating the reaction at 75 °C for 1 min, cooling it back to 42 °C, adding 5.5 μl of RT mixture (2 μl of 5 M betaine, 0.5 μl of 20 μM TSO, 0.6 μl of 100 mM MgCl_2_, 0.8 μl of 25 mM dNTP mixture, 0.6 μl of 10X RT buffer, 0.4 μl of 100 mM DTT, 0.3 μl of 0.1% Triton X-100, 0.25 μl of 200 U/μl superscript II, and 0.05 μl of 20U/μl SUPERase In) to the reaction, and finally incubating the mixture at 42 °C for 90 min and 70 °C for 15 min. If further preamplification was required, the cDNA was added to 40 μl of preamplification mixture (10 μl of 5X KAPAHiFi Fidelity Buffer with MgCl_2_, 1 μl of 10 mM dNTP mixture, 1 μl of 10 μM PCRIIA, 1 μl of 10 μM FC-121-1030, 3 μl of DMSO, 1 unit of KAPAHiFi DNA Polymerase, and 23 μl of water). The thermal cyclings were performed with initial denaturation at 98 °C for 1 min followed by cycles at 98 °C for 10 s, 65 °C for 15 s, and 72 °C for 1 min, with a final extension at 72 °C for 10 min.

### DNA purification with PEG/NaCl

The cDNA from RT reaction/preamplification or the eluted DNA from size selection was concentrated with carboxylated beads and PEG/NaCl [35]. Briefly, 1 μl of magnetic beads and 3 volumes of PEG/NaCl solution were mixed with the solution containing DNA. The mixtures were left at ambient temperature for 15 min. After being washed two times with freshly prepared 80% ethanol, the beads were dried at ambient temperature for 15 min on a magnetic stand and eluted with 0.1% Triton X-100 for another 15 min at ambient temperature.

### Denaturing PAGE and silver staining of gels

The polyacrylamide gels were made of 1X Tris-borate-EDTA (TBE) plus 8 M urea, and TBE PAGE was performed in a 55 °C water bath to ensure the denaturing of the DNA samples. The nondenaturing PAGE was performed by using polyacrylamide gels without urea and running the gel at ambient temperature. Silver staining of the gels was based on an established protocol [36] with minor modifications. Briefly, the gel was fixed in 10% ethanol/0.5% acetic acid for 5 min. After being washed twice with water, the gel was stained with 0.15% silver nitrate/0.15% formaldehyde for 10 min. After again being washed twice with water, the gel was developed in 1.5% sodium hydroxide/0.3% formaldehyde for 10 min, and the reaction was finally stopped with 10% ethanol/0.5% acetic acid. The entire procedure was conducted at ambient temperature.

### Library preparation and high-throughput sequencing

After preamplification and concentration with PEG/NaCl, the cDNA was run in 6% denaturing PAGE and stained in a 1X TBE buffer containing 1X SYBR Gold for 20 min. After visualization and size selection, the gel pieces were crushed by being spun through a 0.5-ml microtube with a pinhole at the bottom and soaked in 300 μl of 10 mM Tris 8.0, 300 mM NaCl, and 1 mM EDTA at ambient temperature overnight. After concentration with PEG/NaCl, the eluted cDNA was appended with P5 and P7 adapters by 10 cycles of PCR with KAPAHiFi DNA polymerase (conditions identical to preamplification, except that P5PCRIIA and N7xx were used as primers). After another round of PEG/NaCl to remove the primers, equal amounts of the indexed libraries were mixed for sequencing with the Illumina Hiseq 2500 system.

### Culture, library preparation, and sequencing of 293FT and 293T cells

The cells were cultured in DMEM/F12 with 10% fetal calf serum (FCS, ThermoFisher). One hundred cells were lysed (293FT) or sorted directly (293T) into 2 μl lysis buffer (0.1% Triton X-100 with 100 μg/ml of proteinase K) and subject to STA (15-cycle preamplification). After PEG/NaCl purification, the eluted libraries underwent denaturing PAGE (12%) and size selection (location detailed in Additional file 4: Figure S2E) against 21- and 27-mer mock libraries prepared with the R2, instead of i7, adapter to avoid carryover contamination. After gel purification and 10 cycles of adapter-attachment PCR, the PEG/NaCl-purified libraries were sequenced with the Illumina Hiseq 2500 system with low-complexity option to avoid potential biased base composition during the first few cycles of sequencing.

### Bioinformatics

The raw sequencing data were processed through FASTQ Groomer using the basic options on the Galaxy website [37]. Reads with more than eight As at the 3’ ends were selected for further analyses, and the A tails were clipped away using FASTQ/A Clipper [38]. The filtered reads were aligned to the human genome assembly version GRCh38 using STAR aligner [39] with the following parameters: --outSAMmultNmax 1 --outMultimapperOrder Random --outFilterMatchNminOverLread 0.95 --outFilterType BySJout --outSAMtype BAM SortedByCoordinate --outFilterMatchNmin 18 --outWigType bedGraph --outWigStrand Stranded --outWigNorm RPM --outWigReferencesPrefix chr --genomeSAsparseD 2. Gene body coverage was performed with the RSeQC package [40]. The aligned reads were assigned to the GENCODE v22 features and counted with the BEDTools [41] “multicov” function while the strandedness was taken into account. All miRNA quantifications, except the percentages of mature miRNA to total miRNA reads at the bottom of Additional file 2: Table S1 and PCA of cell-type segregation in Fig. 4f, were based on the miRNA subset in GENCODE v22 (only primary miRNA transcripts were annotated in the database). To count mature RNA reads (Additional file 2: Table S1 and Fig. 4f), the nonmature miRNA feature was obtained by subtracting the mature “miRNA” feature from the “miRNA_primary_transcript” one in the miRBase v21 database with the BEDTools “subtract” function. Reads aligned to the nonmature miRNA feature were removed with the BEDTools “intersect –v” function. The subtracted reads were then used to get mature miRNA counts by the mature “miRNA” feature in the miRBase database. BioVenn was used to show the Venn diagrams [42]. The differential expression and normalization of counts among the samples were performed with DESeq2 [43]. The reads were visualized with UCSC track hubs [44]. Positive and negative values on the *x*-axis represented reads aligned to the forward and reverse strands, respectively. The peaks on the reverse strand were shown with a fainter color. Default parameters were used for presenting specific data using R tools [45] unless otherwise specified. The raw sequencing data of all 18 samples, RNA counts by GENCODE v22, mature miRNA counts by miRBase v21, and the differential-expression analyses of protein-coding genes (PSC vs 293) and of miRNAs (PSC vs 293 and PSC vs END) with DESeq2 are available at GEO (GSE93672, https://www.ncbi.nlm.nih.gov/geo/query/acc.cgi?acc=GSE93672).

### qPCR analysis

The fluorescence levels of SYBR Green during the PCR (initial denaturation at 94 °C for 1 min followed by 40 cycles at 94 °C for 5 s and at 60 °C for 1 min) were collected and analyzed with the “qpcR” package [46] with the following parameters: model = “l5”, type.eff = “mean.pair.” The confidence intervals from the permutation analysis were exported for statistical evaluation and plotting. For miR-196b that was undetectable by the qPCR in hiPSCs (Fig. 2h and i), the cpD2 was set to 50, and the amplification efficiencies of miR-196b in 293FT were used for calculating the fold changes. The expression of PSMD4 (Fig. 3) or RNAU6 (Fig. 2) was used as the reference to normalize expression across samples.

## Additional files

Additional file 1:
**Figure S1.** Optimizing the conditions for single-tube amplification (*STA*) of RNA. (A) Expanded view of the denaturing PAGE in Fig. 1f demonstrating the unspecific amplification products from the control cDNA (*Control*, *asterisks*) vs the clean backgrounds from the preamplified cDNA (*PreAmp*). (B) Quantification of preamplified cDNA in Fig. 1f by qPCR. Identical amounts of PreAmp eluents were used as input for PCR in Fig. 1f and qPCR here. *cpD2* and *Eff*: cycle threshold and efficiency, respectively, using the qpcR package. (C) Denaturing PAGE (6%) of the preamplified cDNA in Fig. 1g. One-fifth of the purified, preamplified (21 cycles) cDNAs was loaded in each lane. (D) Expanded view of the denaturing PAGE in Fig. 1g showing the unspecific amplified products (*asterisks*) in the -RT, -PAP, and no-cell controls. (E) Quantification of the spiked-in small-RNA oligos (*RNA*_*27*_) in Fig. 1h by qPCR. Identical amounts of PreAmp eluents were used as input for PCR in Fig. 1g and qPCR here. The expression of endogenous miR-let7a in the hESC line served as a loading control. *cpD2* and *Eff*: cycle threshold and efficiency, respectively, using the qpcR package. (JPG 3549 kb)
Additional file 2:
**Table S1.** Summary of the sequencing results. The alignments against the GRCh38 genome assembly (Aligned Reads) were counted for exon reads (exon) and transcript reads based on GENCODE v22. Intronic counts (intron) were defined by transcript counts minus exon ones. Nontranscript reads were used to obtain tRNA counts (tRNA) based on the tRNA database of GENCODE v22. Nontranscript and non-tRNA reads were used for counts on repetitive sequences (repeats) based on RepeatMasker. Those not belonging to any category were defined as unannotated reads (unannotated). The counting of exonic features was based on the “gene_type” attribute in GENCODE v22. The percentages of mature miRNA reads were defined by reads aligned exclusively to the mature “miRNA” feature divided by reads aligned to the “miRNA_primary_transcript” feature of miRBase v21. (DOCX 42 kb)
Additional file 3:
**Table S2.** The locations and identities of the top 40 abundant reads belonging to the unannotated category after feature assignment. The nontranscript, non-tRNA (GENCODE v22), and nonrepeat (RepeatMasker) reads of TP_100N_, CP_100N_, and CP_100W_ were pooled together and merged into genome coordinates with the “bedtools merge” function. The numbers of reads on each coordinate were counted. The percentages (%) were defined as the counts on each coordinate divided by the summed counts of all coordinates. The features were searched by the order of human mRNA, Expressed Sequence Tag (*EST*), and Basic Local Alignment Search Tool (*BLAST*) against the human transcriptome. (DOCX 13 kb)
Additional file 4:
**Figure S2.** High-throughput profiling of hPSC and 293-cell transcriptomes based on STA. (A) Visualization of reads in the UCSC Genome Browser. Each curve represents reads per million (*RPM*)-normalized wiggle output of the libraries against the GRCh38 genome assembly. (B) Scatter plot showing the correlation of miRNA levels between samples. *Upper*: CP_100_N (Ch8 hES cells) vs TP_100_N (TW1 hES cells); *lower*: Solexa vs 454 sequencing data from an hESC sample in the literature. (C) and (D) represent the Venn diagrams (*upper*) and scatter plots (*lower*) for the miRNA levels of CP_100_N (C) and CP_100_W (D) against the reference Solexa data. *Blue* and *green* indicate the top 10 highest expressers in the respective libraries, and *red* indicates the genes that were in the top 10 in both libraries. Each value of the reference samples was multiplied by 10,000 in (B), (C), and (D) because only normalized data were available with the reference samples. (E) Representative denaturing PAGE (12%) of cDNA libraries from RNA oligos, a no-cell control, and 100 293FT cells. One pmol of 21-mer (*RNA*_*21*_) and 27-mer (*RNA*_*27*_) RNAs were subject to polyadenylation, TS reaction, and direct PEG/NaCl purification. R2T20V, instead of i7T20V, was used for lanes 1 and 3 during TS reaction. One-hundredth of the eluents was amplified (20 cycles of PCR) with the following primer combinations: lane 1, SMART21 + R2; lane 2, SMART21 + FC-121-1030; lane 3, SMART27 + R2; lane 4, SMART27 + FC-121-1030. Lane 5 represents combined loading of lanes 1 and 3 to serve as a mock cDNA library for size selection. R2-based mock libraries were used to avoid contamination during sequencing. Lanes 6 and 7, respectively, represent STA libraries (15-cycle preamplification) from a no-cell control and 100 cells of 293FT. *H*, *M*, *L*, *arrow* (RNA_27_ library location) and *arrowhead* (RNA_21_ library location) indicate the size ranges harvested for library preparation. (F) Venn diagram showing the overlap of miRNAs identified (counts >2.9 RPM to account for different depth of coverage among samples) in 293FT_M_ and those in two independent reference sRNA-Seq data (SRX556516 and SRX763661) sources from 293 cells. (G) Scatter plot demonstrating the association of rlog-normalized (*rld*) miRNA quantifications of 293 cells between libraries. *Left*: sequencing data with STA (293FT_M_) vs reference SRX556516 sRNA-Seq data; *right*: STA-based sequencing data between 293FT (293FT_M_) and 293T (293T_M_) cell lines. Identical genome alignment (GRCh38) and feature assignment (GENCODE v22) were performed, and miRNAs (summed counts >1 across all samples) were used for rlog transformation (blind = TRUE) with DESeq2. (H) Scatter plot demonstrating the association of rlog-normalized (*rld*) lincRNA quantifications of 293 cells between the 293FT_M_ library vs the reference SRX763661 (*left*) and SRX556516 (*right*) sequencing data. Identical genome alignment (GRCh38) and feature assignment (GENCODE v22) were performed, and all lincRNAs (raw counts >0) were used for rlog transformation (blind = TRUE) with DESeq2. (I) Scatter plots of average rlog-transformed counts from three PSC (TP100N, CP100N, and CP100W, *y*-axis) and all six 293T (*x*-axis) samples. Genes with counts >1 across all nine samples were included for rlog transformation and differential-expression analysis with DESeq2. Genes with adjusted *p* value <0.05 are highlighted *blue* (enriched in PSC) or *black* (enriched in 293T cells). *Left*: protein-coding subset; *right*: miRNA subset. (JPG 15934 kb)
Additional file 5:
**Figure S3.** Profiling the differential expression of RNA between hPSCs and the differentiated endothelial cells using STA. (A) Gene body coverage chart of all aligned reads from 100-cell endothelial libraries against all transcripts in GENCODE v22. (B) Unsupervised PCA based on the expression of miRNA of 100 hESCs and the differentiated endothelial cells. miRNAs (gene type = “miRNA”, GENCODE v22) with summed counts >20 across six samples were rlog-transformed with DESeq2 (blind = TRUE), and the output was subject to PCA with default parameters. (C) Unsupervised heat map of the data in (B). The top 150 variable miRNAs of the rlog-transformed counts in (B) served as the input for heatmap3 analysis with default parameters. *Blue* and *red clusters* indicated gene enriched in hESCs and endothelial cells, respectively. (D) Visualization of the miRNA peaks of the six 100-cell samples in the UCSC Genome Browser. Each curve represents RPM-normalized wiggle output of the libraries against the GRCh38 genome assembly. (E) Denaturing PAGE (12%) of the rest of the semi-quantitative PCR products in Fig. 3f. (JPG 10181 kb)
Additional file 6:
**Figure S4.** Probing the detection limit with 10 to single cells. (A) Gene body coverage chart of all aligned reads from six low-input libraries against all transcripts in the GENCODE v22. (B) The supervised heat map for the expression of miRNAs of the 12 PSC and END samples. miRNAs (GENCODE v22; summed count across all samples >1) from the samples were used for differential-expression analysis with DESeq2. The rlog-transformed counts with lowest *p* values (1000) were ranked by log2 fold changes and served as input for heatmap3 without rearranging column and row dendrograms. (C) Supervised PCA of the total RNA from the 12 PSC and END samples by the principal components derived from 100 cells in Fig. 3b. The PCA derived from the analysis in Fig. 3b was applied to the rlog-transformed counts (GENCODE v22; summed counts across all samples >20) of the 12 samples. (D) Scatter plots of rld_miRNA_ from individual samples of endothelial cells vs the averaged rld_miRNA_ of all six hPSC samples. Only miRNAs with summed counts >20 across the 12 PSC and END samples were included for DESeq2 analysis. The *colored dots* represent miRNAs enriched in hPSCs (*blue*) or endothelial cells (*red*) as defined in Fig. 3d. The *red dots* were transformed into *squares* when the values of (rld – average rld)/log10 (10 + average rld) were less than or equal to 0.4. The ratio of *red squares* over total *red* (*square* + dots) is indicated in the *upper left corner*. Exemplary aberrant expressers in low-input samples are highlighted with *black borders*. (E) Visualization of the miRNA peaks in the UCSC Genome Browser. Each curve represents RPM-normalized wiggle output of the libraries against the GRCh38 genome assembly. (F) Scatter plot showing the correlation of miRNA expression between libraries prepared from narrow (*N*) and wide (*W*) gel slices. Log2-transformed raw counts of miRNAs (gene type = “miRNA”, GENCODE v22) were plotted for Ch8 hESCs (*left*; CP_100_W vs CP_100_N) and their differentiated endothelial cells (*right*; CE_100_W vs CE_100_N) of the 100-cell sequencing data in Fig. 3. *Blue* and *green* indicate the top 10 highest expressers in the respective libraries, and *red* indicates the genes that were in the top 10 in both libraries. (G) Scatter plots of rlog-transformed miRNA counts between the 293FT_M_ library and libraries from different gel-slice locations in Additional file 4: Figure S2E. miRNAs (gene type = “miRNA”, GENCODE v22) with summed counts >1 were rlog-transformed with DESeq2 (blind = TRUE), and the transformed values between individual samples were plotted. (JPG 16436 kb)
Additional file 7:
**Table S3.** Primers used for library preparation and PCR. Matched or complementary sequences are either color-coded or underlined to aid identification. (DOCX 23 kb)
