# Supplementary figures and images for: Low-cell-number, single-tube amplification (STA) of total RNA revealed transcriptome changes from pluripotency to endothelium
Source: BMC Biol. 2017 Mar 21;15:22. doi: 10.1186/s12915-017-0359-5 (PMC5360049; doi:10.1186/s12915-017-0359-5)

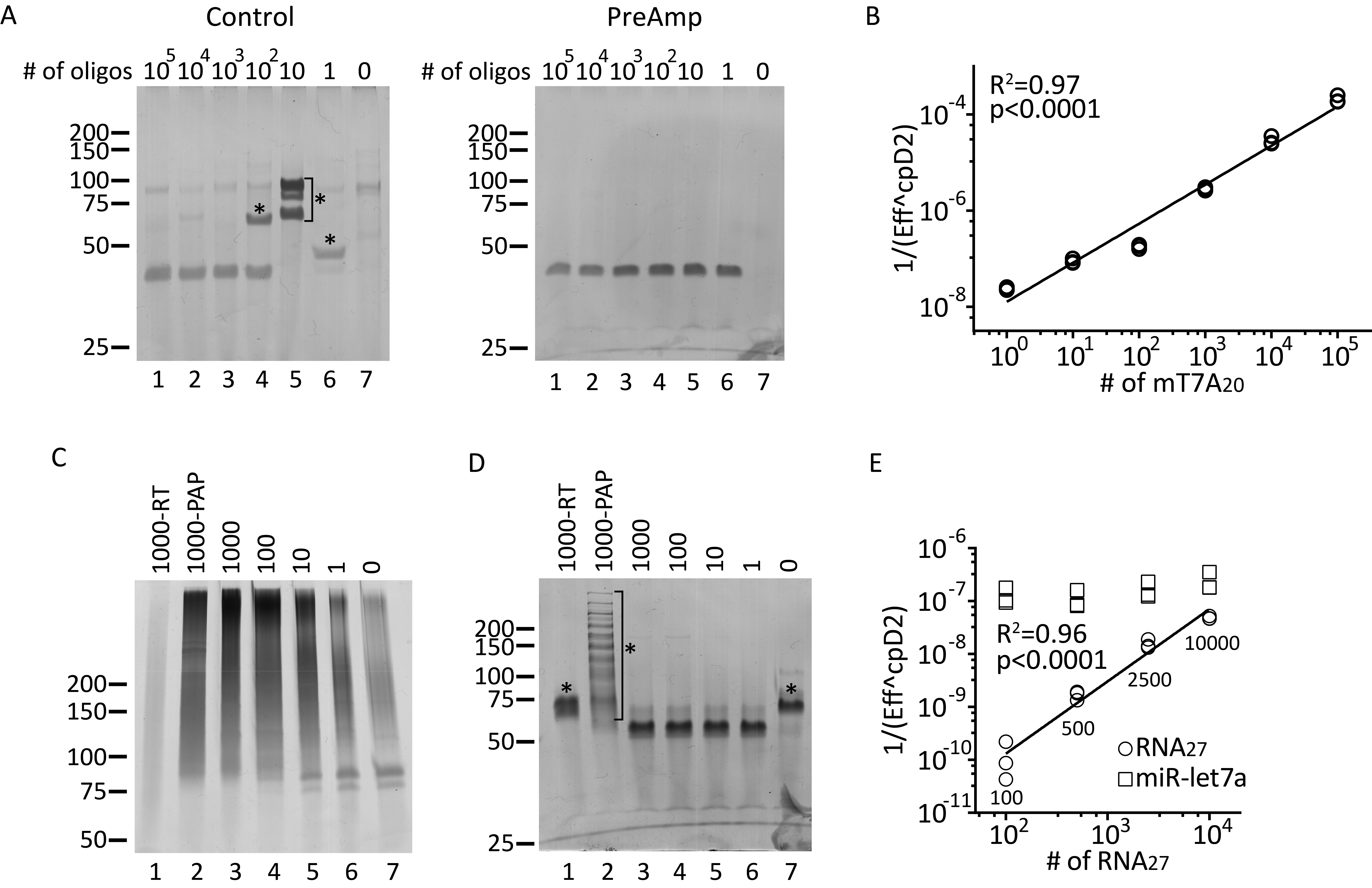

Supplement: Additional file 1: — Figure S1. Optimizing the conditions for single-tube amplification (STA) of RNA. (A) Expanded view of the denaturing PAGE in Fig. 1f demonstrating the unspecific amplification products from the control cDNA (Control, asterisks) vs the clean backgrounds from the preamplified cDNA (PreAmp). (B) Quantification of preamplified cDNA in Fig. 1f by qPCR. Identical amounts of PreAmp eluents were used as input for PCR in Fig. 1f and qPCR here. cpD2 and Eff: cycle threshold and efficiency, respectively, using the qpcR package. (C) Denaturing PAGE (6%) of the preamplified cDNA in Fig. 1g. One-fifth of the purified, preamplified (21 cycles) cDNAs was loaded in each lane. (D) Expanded view of the denaturing PAGE in Fig. 1g showing the unspecific amplified products (asterisks) in the -RT, -PAP, and no-cell controls. (E) Quantification of the spiked-in small-RNA oligos (RNA 27) in Fig. 1h by qPCR. Identical amounts of PreAmp eluents were used as input for PCR in Fig. 1g and qPCR here. The expression of endogenous miR-let7a in the hESC line served as a loading control. cpD2 and Eff: cycle threshold and efficiency, respectively, using the qpcR package. (JPG 3549 kb) [file 12915_2017_359_MOESM1_ESM.jpg]

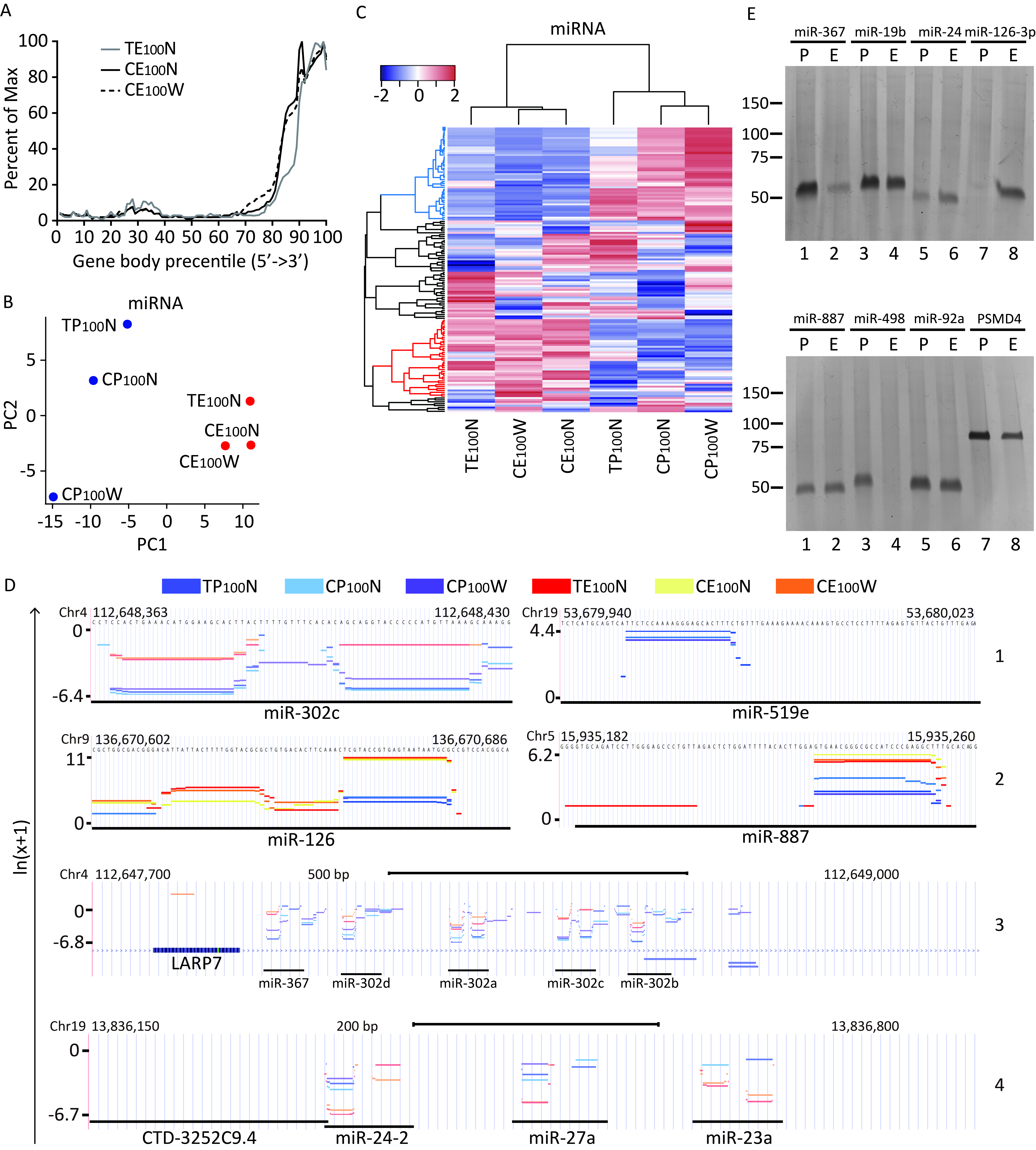

Supplement: Additional file 5: — Figure S3. Profiling the differential expression of RNA between hPSCs and the differentiated endothelial cells using STA. (A) Gene body coverage chart of all aligned reads from 100-cell endothelial libraries against all transcripts in GENCODE v22. (B) Unsupervised PCA based on the expression of miRNA of 100 hESCs and the differentiated endothelial cells. miRNAs (gene type = “miRNA”, GENCODE v22) with summed counts >20 across six samples were rlog-transformed with DESeq2 (blind = TRUE), and the output was subject to PCA with default parameters. (C) Unsupervised heat map of the data in (B). The top 150 variable miRNAs of the rlog-transformed counts in (B) served as the input for heatmap3 analysis with default parameters. Blue and red clusters indicated gene enriched in hESCs and endothelial cells, respectively. (D) Visualization of the miRNA peaks of the six 100-cell samples in the UCSC Genome Browser. Each curve represents RPM-normalized wiggle output of the libraries against the GRCh38 genome assembly. (E) Denaturing PAGE (12%) of the rest of the semi-quantitative PCR products in Fig. 3f. (JPG 10181 kb) [file 12915_2017_359_MOESM5_ESM.jpg]
