# Supplementary material for: Low-cell-number, single-tube amplification (STA) of total RNA revealed transcriptome changes from pluripotency to endothelium
Source: BMC Biol. 2017 Mar 21;15:22. doi: 10.1186/s12915-017-0359-5 (PMC5360049; doi:10.1186/s12915-017-0359-5)
Supplement: Additional file 2: — Table S1. Summary of the sequencing results. The alignments against the GRCh38 genome assembly (Aligned Reads) were counted for exon reads (exon) and transcript reads based on GENCODE v22. Intronic counts (intron) were defined by transcript counts minus exon ones. Nontranscript reads were used to obtain tRNA counts (tRNA) based on the tRNA database of GENCODE v22. Nontranscript and non-tRNA reads were used for counts on repetitive sequences (repeats) based on RepeatMasker. Those not belonging to any category were defined as unannotated reads (unannotated). The counting of exonic features was based on the “gene_type” attribute in GENCODE v22. The percentages of mature miRNA reads were defined by reads aligned exclusively to the mature “miRNA” feature divided by reads aligned to the “miRNA_primary_transcript” feature of miRBase v21. (DOCX 42 kb) [file 12915_2017_359_MOESM2_ESM.docx]

|  | PSC | | | | | | 293 | | | | | |
| --- | --- | --- | --- | --- | --- | --- | --- | --- | --- | --- | --- | --- |
|  | TP100N | CP100N | CP100W | CP10W | TP10N | TP1N | 293FTH | 293FTM | 293FTL | 293FT21 | 293FT27 | 293TM |
| Input Reads | 3955938 | 3020218 | 3035522 | 2078130 | 1742731 | 3510775 | 26308396 | 27717138 | 28442806 | 14403619 | 13217624 | 21981306 |
| Aligned Reads | 1386027 100.0% | 1246686 100.0% | 1515636 100.0% | 1033445 100.0% | 289397 100.0% | 391648 100.0% | 12070117 100.0% | 13499417 100.0% | 1897606 100.0% | 8159447 100.0% | 6222772 100.0% | 11141715 100.0% |
| exon | 246839 17.8% | 144459 11.6% | 176790 11.7% | 108652 10.5% | 50630 17.5% | 54025 13.8% | 2049786 17.0% | 3802051 28.2% | 365708 19.3% | 2266657 27.8% | 1823205 29.3% | 2602053 23.4% |
| intron | 292673 21.1% | 281951 22.6% | 299033 19.7% | 215842 20.9% | 71846 24.8% | 95214 24.3% | 1471802 12.2% | 2623984 19.4% | 406190 21.4% | 1629124 20.0% | 1213680 19.5% | 2591247 23.3% |
| tRNA | 175631 12.7% | 58096 4.7% | 195983 12.9% | 94367 9.1% | 34196 11.8% | 30252 7.7% | 6267701 51.9% | 2155329 16.0% | 145986 7.7% | 744549 9.1% | 1342194 21.6% | 1664744 14.9% |
| repeats | 296778 21.4% | 256450 20.6% | 248404 16.4% | 191453 18.5% | 75325 26.0% | 131288 33.5% | 809545 6.7% | 2272204 16.8% | 455863 24.0% | 1600197 19.6% | 824180 13.2% | 1885513 16.9% |
| unannotated | 374106 27.0% | 505730 40.6% | 595426 39.3% | 423131 40.9% | 57400 19.8% | 80869 20.6% | 1471283 12.2% | 2645849 19.6% | 523859 27.6% | 1918920 23.5% | 1019513 16.4% | 2398158 21.5% |
| Exon Reads | 451600 100.0% | 246786 100.0% | 303790 100.0% | 170741 100.0% | 83164 100.0% | 68544 100.0% | 3145182 100.0% | 6671744 100.0% | 706097 100.0% | 3876165 100.0% | 3116463 100.0% | 4992522 100.0% |
| lincRNA | 16763 3.7% | 4590 1.9% | 7211 2.4% | 4030 2.4% | 4358 5.2% | 3189 4.7% | 211430 6.7% | 195261 2.9% | 20615 2.9% | 105369 2.7% | 83939 2.7% | 144063 2.9% |
| miRNA | 23951 5.3% | 27378 11.1% | 45774 15.1% | 40917 24.0% | 5581 6.7% | 2278 3.3% | 79142 2.5% | 345814 5.2% | 43430 6.2% | 266912 6.9% | 90106 2.9% | 96201 1.9% |
| Mt_rRNA | 4757 1.1% | 2581 1.0% | 4395 1.4% | 2913 1.7% | 1288 1.5% | 1016 1.5% | 77735 2.5% | 92320 1.4% | 7750 1.1% | 42814 1.1% | 51408 1.6% | 83353 1.7% |
| Mt_tRNA | 28361 6.3% | 10670 4.3% | 11612 3.8% | 4444 2.6% | 3841 4.6% | 2900 4.2% | 634361 20.2% | 1110711 16.6% | 61299 8.7% | 486968 12.6% | 750723 24.1% | 464041 9.3% |
| protein_coding | 185736 41.1% | 143345 58.1% | 146817 48.3% | 74967 43.9% | 33450 40.2% | 35859 52.3% | 852863 27.1% | 2130939 31.9% | 317247 44.9% | 1245156 32.1% | 888312 28.5% | 2282565 45.7% |
| rRNA | 22831 5.1% | 12843 5.2% | 29558 9.7% | 13604 8.0% | 6228 7.5% | 3517 5.1% | 145557 4.6% | 350555 5.3% | 39723 5.6% | 403563 10.4% | 101011 3.2% | 436230 8.7% |
| snoRNA | 97685 21.6% | 25859 10.5% | 31878 10.5% | 14970 8.8% | 16884 20.3% | 11568 16.9% | 407270 12.9% | 1199063 18.0% | 84774 12.0% | 507979 13.1% | 665943 21.4% | 723358 14.5% |
| snRNA | 21412 4.7% | 2510 1.0% | 3316 1.1% | 2216 1.3% | 2295 2.8% | 1694 2.5% | 86877 2.8% | 300659 4.5% | 35184 5.0% | 206208 5.3% | 93353 3.0% | 153811 3.1% |
| 3prime_overlapping_ncrna | 31 0.0% | 6 0.0% | 12 0.0% | 10 0.0% | 0 0.0% | 32 0.0% | 81 0.0% | 177 0.0% | 17 0.0% | 141 0.0% | 95 0.0% | 339 0.0% |
| antisense | 4134 0.9% | 1286 0.5% | 1765 0.6% | 1221 0.7% | 818 1.0% | 553 0.8% | 245098 7.8% | 95900 1.4% | 8869 1.3% | 34193 0.9% | 43881 1.4% | 82415 1.7% |
| IG_C_gene | 0 0.0% | 0 0.0% | 0 0.0% | 0 0.0% | 0 0.0% | 0 0.0% | 0 0.0% | 13 0.0% | 10 0.0% | 0 0.0% | 0 0.0% | 14 0.0% |
| IG_C_pseudogene | 0 0.0% | 0 0.0% | 0 0.0% | 0 0.0% | 0 0.0% | 0 0.0% | 0 0.0% | 0 0.0% | 0 0.0% | 0 0.0% | 0 0.0% | 0 0.0% |
| IG_D_gene | 0 0.0% | 0 0.0% | 0 0.0% | 0 0.0% | 0 0.0% | 0 0.0% | 0 0.0% | 0 0.0% | 1 0.0% | 0 0.0% | 0 0.0% | 0 0.0% |
| IG_J_gene | 0 0.0% | 0 0.0% | 0 0.0% | 0 0.0% | 0 0.0% | 0 0.0% | 0 0.0% | 0 0.0% | 0 0.0% | 0 0.0% | 0 0.0% | 0 0.0% |
| IG_J_pseudogene | 0 0.0% | 0 0.0% | 0 0.0% | 0 0.0% | 0 0.0% | 0 0.0% | 0 0.0% | 0 0.0% | 0 0.0% | 0 0.0% | 0 0.0% | 0 0.0% |
| IG_V_gene | 0 0.0% | 0 0.0% | 0 0.0% | 4 0.0% | 0 0.0% | 0 0.0% | 0 0.0% | 1 0.0% | 0 0.0% | 1 0.0% | 0 0.0% | 2 0.0% |
| IG_V_pseudogene | 0 0.0% | 0 0.0% | 2 0.0% | 0 0.0% | 0 0.0% | 0 0.0% | 0 0.0% | 1 0.0% | 0 0.0% | 1 0.0% | 0 0.0% | 8 0.0% |
| macro_lncRNA | 1 0.0% | 0 0.0% | 0 0.0% | 4 0.0% | 0 0.0% | 5 0.0% | 25 0.0% | 38 0.0% | 11 0.0% | 7 0.0% | 11 0.0% | 74 0.0% |
| misc_RNA | 2740 0.6% | 1509 0.6% | 3989 1.3% | 2650 1.6% | 771 0.9% | 274 0.4% | 133910 4.3% | 119085 1.8% | 8750 1.2% | 59809 1.5% | 51841 1.7% | 77110 1.5% |
| non_coding | 263 0.1% | 128 0.1% | 269 0.1% | 156 0.1% | 23 0.0% | 13 0.0% | 21016 0.7% | 18002 0.3% | 1230 0.2% | 10513 0.3% | 8190 0.3% | 5491 0.1% |
| polymorphic_pseudogene | 0 0.0% | 1 0.0% | 0 0.0% | 5 0.0% | 0 0.0% | 38 0.1% | 0 0.0% | 57 0.0% | 9 0.0% | 37 0.0% | 43 0.0% | 1 0.0% |
| processed_pseudogene | 5673 1.3% | 2585 1.0% | 2510 0.8% | 1139 0.7% | 1008 1.2% | 1018 1.5% | 62919 2.0% | 128151 1.9% | 11128 1.6% | 91293 2.4% | 61206 2.0% | 70364 1.4% |
| processed_transcript | 27547 6.1% | 8342 3.4% | 9949 3.3% | 4673 2.7% | 3715 4.5% | 2684 3.9% | 119860 3.8% | 459864 6.9% | 49712 7.0% | 330966 8.5% | 179304 5.8% | 243496 4.9% |
| pseudogene | 8 0.0% | 5 0.0% | 6 0.0% | 3 0.0% | 1 0.0% | 0 0.0% | 24 0.0% | 85 0.0% | 14 0.0% | 29 0.0% | 15 0.0% | 97 0.0% |
| ribozyme | 6349 1.4% | 1665 0.7% | 1662 0.5% | 1189 0.7% | 2478 3.0% | 1140 1.7% | 10817 0.3% | 71483 1.1% | 10559 1.5% | 61269 1.6% | 24763 0.8% | 87947 1.8% |
| scaRNA | 621 0.1% | 125 0.1% | 165 0.1% | 90 0.1% | 39 0.0% | 51 0.1% | 5668 0.2% | 13684 0.2% | 1433 0.2% | 6095 0.2% | 6578 0.2% | 5733 0.1% |
| sense_intronic | 1753 0.4% | 414 0.2% | 1586 0.5% | 679 0.4% | 133 0.2% | 104 0.2% | 45282 1.4% | 26800 0.4% | 1885 0.3% | 9412 0.2% | 9613 0.3% | 18423 0.4% |
| sense_overlapping | 61 0.0% | 23 0.0% | 36 0.0% | 36 0.0% | 6 0.0% | 7 0.0% | 489 0.0% | 1216 0.0% | 114 0.0% | 592 0.0% | 581 0.0% | 1283 0.0% |
| sRNA | 0 0.0% | 0 0.0% | 0 0.0% | 2 0.0% | 0 0.0% | 0 0.0% | 22 0.0% | 2 0.0% | 0 0.0% | 0 0.0% | 2 0.0% | 2 0.0% |
| TEC | 180 0.0% | 227 0.1% | 129 0.0% | 97 0.1% | 59 0.1% | 40 0.1% | 195 0.0% | 2178 0.0% | 826 0.1% | 1706 0.0% | 334 0.0% | 3414 0.1% |
| transcribed_processed_pseudogene | 215 0.0% | 142 0.1% | 188 0.1% | 118 0.1% | 42 0.1% | 43 0.1% | 1558 0.0% | 2173 0.0% | 269 0.0% | 1011 0.0% | 1055 0.0% | 3938 0.1% |
| transcribed_unitary_pseudogene | 0 0.0% | 0 0.0% | 0 0.0% | 0 0.0% | 0 0.0% | 0 0.0% | 0 0.0% | 1 0.0% | 0 0.0% | 0 0.0% | 0 0.0% | 1 0.0% |
| ranscribed_unprocessed_pseudogen | 238 0.1% | 177 0.1% | 231 0.1% | 195 0.1% | 62 0.1% | 247 0.4% | 1175 0.0% | 2910 0.0% | 481 0.1% | 1449 0.0% | 1223 0.0% | 3939 0.1% |
| translated_processed_pseudogene | 0 0.0% | 0 0.0% | 0 0.0% | 0 0.0% | 0 0.0% | 0 0.0% | 0 0.0% | 0 0.0% | 0 0.0% | 0 0.0% | 0 0.0% | 0 0.0% |
| translated_unprocessed_pseudogene | 0 0.0% | 0 0.0% | 0 0.0% | 0 0.0% | 0 0.0% | 0 0.0% | 0 0.0% | 0 0.0% | 0 0.0% | 0 0.0% | 0 0.0% | 0 0.0% |
| TR_C_gene | 0 0.0% | 0 0.0% | 0 0.0% | 0 0.0% | 1 0.0% | 0 0.0% | 0 0.0% | 1 0.0% | 0 0.0% | 0 0.0% | 0 0.0% | 0 0.0% |
| TR_D_gene | 0 0.0% | 0 0.0% | 0 0.0% | 0 0.0% | 0 0.0% | 0 0.0% | 0 0.0% | 0 0.0% | 0 0.0% | 0 0.0% | 0 0.0% | 0 0.0% |
| TR_J_gene | 0 0.0% | 0 0.0% | 0 0.0% | 0 0.0% | 0 0.0% | 0 0.0% | 0 0.0% | 2 0.0% | 0 0.0% | 1 0.0% | 0 0.0% | 1 0.0% |
| TR_J_pseudogene | 0 0.0% | 0 0.0% | 0 0.0% | 0 0.0% | 0 0.0% | 0 0.0% | 0 0.0% | 0 0.0% | 0 0.0% | 0 0.0% | 0 0.0% | 0 0.0% |
| TR_V_gene | 0 0.0% | 0 0.0% | 0 0.0% | 1 0.0% | 23 0.0% | 41 0.1% | 0 0.0% | 2 0.0% | 2 0.0% | 1 0.0% | 3 0.0% | 1 0.0% |
| TR_V_pseudogene | 0 0.0% | 0 0.0% | 0 0.0% | 0 0.0% | 7 0.0% | 0 0.0% | 0 0.0% | 0 0.0% | 0 0.0% | 0 0.0% | 0 0.0% | 0 0.0% |
| unitary_pseudogene | 8 0.0% | 13 0.0% | 24 0.0% | 4 0.0% | 0 0.0% | 24 0.0% | 161 0.0% | 413 0.0% | 84 0.0% | 110 0.0% | 216 0.0% | 545 0.0% |
| unprocessed_pseudogene | 282 0.1% | 275 0.1% | 265 0.1% | 244 0.1% | 53 0.1% | 209 0.3% | 1643 0.1% | 4168 0.1% | 670 0.1% | 2550 0.1% | 2712 0.1% | 4262 0.1% |
| vaultRNA | 0 0.0% | 87 0.0% | 441 0.1% | 160 0.1% | 0 0.0% | 0 0.0% | 4 0.0% | 15 0.0% | 1 0.0% | 10 0.0% | 2 0.0% | 0 0.0% |
| mature_miRNA (%) | 58.3% | 53.5% | 38.6% | 29.8% | 57.3% | 32.8% | 28.0% | 80.8% | 87.3% | 88.0% | 65.4% | 72.1% |

|  | END | | | | | |
| --- | --- | --- | --- | --- | --- | --- |
|  | TE100N | CE100N | CE100W | CE10W | TE10N | TE1N |
| Input Reads | 2095673 | 1032522 | 3388238 | 1868119 | 1543245 | 1704007 |
| Aligned Reads | 511223 100.0% | 329648 100.0% | 737889 100.0% | 552846 100.0% | 217796 100.0% | 239288 100.0% |
| exon | 124770 24.4% | 56374 17.1% | 132779 18.0% | 63102 11.4% | 43114 19.8% | 38551 16.1% |
| intron | 104315 20.4% | 67227 20.4% | 115167 15.6% | 71837 13.0% | 54513 25.0% | 43288 18.1% |
| tRNA | 69005 13.5% | 99907 30.3% | 267269 36.2% | 246418 44.6% | 15489 7.1% | 60524 25.3% |
| repeats | 128716 25.2% | 39337 11.9% | 88557 12.0% | 55457 10.0% | 65297 30.0% | 51354 21.5% |
| unannotated | 84417 16.5% | 66803 20.3% | 134117 18.2% | 116032 21.0% | 39383 18.1% | 45571 19.0% |
| Exon Reads | 215787 100.0% | 92758 100.0% | 212626 100.0% | 100507 100.0% | 64131 0.0% | 41296 100.0% |
| lincRNA | 9037 4.2% | 2725 2.9% | 9083 4.3% | 5676 5.6% | 3211 5.0% | 1615 3.9% |
| miRNA | 24109 11.2% | 6316 6.8% | 19910 9.4% | 3783 3.8% | 14475 22.6% | 1461 3.5% |
| Mt_rRNA | 2323 1.1% | 971 1.0% | 2230 1.0% | 1175 1.2% | 902 1.4% | 436 1.1% |
| Mt_tRNA | 13488 6.3% | 5361 5.8% | 12076 5.7% | 7321 7.3% | 3010 4.7% | 3234 7.8% |
| protein_coding | 78921 36.6% | 35483 38.3% | 76300 35.9% | 42180 42.0% | 21863 34.1% | 21221 51.4% |
| rRNA | 16350 7.6% | 14683 15.8% | 31973 15.0% | 15869 15.8% | 3777 5.9% | 3537 8.6% |
| snoRNA | 44242 20.5% | 15648 16.9% | 33621 15.8% | 10966 10.9% | 8971 14.0% | 5118 12.4% |
| snRNA | 7522 3.5% | 3959 4.3% | 7728 3.6% | 4162 4.1% | 1519 2.4% | 1256 3.0% |
| 3prime_overlapping_ncrna | 10 0.0% | 4 0.0% | 18 0.0% | 0 0.0% | 16 0.0% | 8 0.0% |
| antisense | 2017 0.9% | 473 0.5% | 1532 0.7% | 1139 1.1% | 535 0.8% | 246 0.6% |
| IG_C_gene | 0 0.0% | 0 0.0% | 0 0.0% | 0 0.0% | 0 0.0% | 0 0.0% |
| IG_C_pseudogene | 0 0.0% | 0 0.0% | 0 0.0% | 0 0.0% | 0 0.0% | 0 0.0% |
| IG_D_gene | 0 0.0% | 0 0.0% | 0 0.0% | 0 0.0% | 0 0.0% | 0 0.0% |
| IG_J_gene | 0 0.0% | 0 0.0% | 0 0.0% | 0 0.0% | 0 0.0% | 0 0.0% |
| IG_J_pseudogene | 0 0.0% | 0 0.0% | 0 0.0% | 0 0.0% | 0 0.0% | 0 0.0% |
| IG_V_gene | 5 0.0% | 1 0.0% | 4 0.0% | 0 0.0% | 0 0.0% | 43 0.1% |
| IG_V_pseudogene | 0 0.0% | 0 0.0% | 0 0.0% | 0 0.0% | 0 0.0% | 0 0.0% |
| macro_lncRNA | 0 0.0% | 0 0.0% | 0 0.0% | 0 0.0% | 0 0.0% | 0 0.0% |
| misc_RNA | 2741 1.3% | 1709 1.8% | 4744 2.2% | 2471 2.5% | 1130 1.8% | 633 1.5% |
| non_coding | 203 0.1% | 157 0.2% | 271 0.1% | 152 0.2% | 22 0.0% | 25 0.1% |
| polymorphic_pseudogene | 0 0.0% | 0 0.0% | 0 0.0% | 0 0.0% | 3 0.0% | 0 0.0% |
| processed_pseudogene | 2650 1.2% | 797 0.9% | 1557 0.7% | 847 0.8% | 553 0.9% | 503 1.2% |
| processed_transcript | 8151 3.8% | 3036 3.3% | 7165 3.4% | 3298 3.3% | 2166 3.4% | 1528 3.7% |
| pseudogene | 0 0.0% | 2 0.0% | 1 0.0% | 0 0.0% | 0 0.0% | 0 0.0% |
| ribozyme | 3204 1.5% | 915 1.0% | 3123 1.5% | 684 0.7% | 1592 2.5% | 164 0.4% |
| scaRNA | 181 0.1% | 93 0.1% | 210 0.1% | 113 0.1% | 55 0.1% | 44 0.1% |
| sense_intronic | 278 0.1% | 233 0.3% | 566 0.3% | 319 0.3% | 83 0.1% | 61 0.1% |
| sense_overlapping | 52 0.0% | 29 0.0% | 51 0.0% | 18 0.0% | 42 0.1% | 44 0.1% |
| sRNA | 0 0.0% | 0 0.0% | 0 0.0% | 0 0.0% | 0 0.0% | 0 0.0% |
| TEC | 87 0.0% | 15 0.0% | 73 0.0% | 60 0.1% | 44 0.1% | 44 0.1% |
| transcribed_processed_pseudogene | 40 0.0% | 37 0.0% | 59 0.0% | 36 0.0% | 10 0.0% | 37 0.1% |
| transcribed_unitary_pseudogene | 0 0.0% | 0 0.0% | 0 0.0% | 0 0.0% | 0 0.0% | 0 0.0% |
| ranscribed_unprocessed_pseudogen | 114 0.1% | 23 0.0% | 99 0.0% | 151 0.2% | 126 0.2% | 6 0.0% |
| translated_processed_pseudogene | 0 0.0% | 0 0.0% | 0 0.0% | 0 0.0% | 0 0.0% | 0 0.0% |
| translated_unprocessed_pseudogene | 0 0.0% | 0 0.0% | 0 0.0% | 0 0.0% | 0 0.0% | 0 0.0% |
| TR_C_gene | 0 0.0% | 0 0.0% | 0 0.0% | 0 0.0% | 0 0.0% | 0 0.0% |
| TR_D_gene | 0 0.0% | 0 0.0% | 0 0.0% | 0 0.0% | 0 0.0% | 0 0.0% |
| TR_J_gene | 0 0.0% | 0 0.0% | 0 0.0% | 0 0.0% | 0 0.0% | 0 0.0% |
| TR_J_pseudogene | 0 0.0% | 0 0.0% | 0 0.0% | 0 0.0% | 0 0.0% | 0 0.0% |
| TR_V_gene | 4 0.0% | 0 0.0% | 0 0.0% | 0 0.0% | 0 0.0% | 0 0.0% |
| TR_V_pseudogene | 0 0.0% | 0 0.0% | 0 0.0% | 0 0.0% | 0 0.0% | 0 0.0% |
| unitary_pseudogene | 1 0.0% | 0 0.0% | 13 0.0% | 0 0.0% | 0 0.0% | 1 0.0% |
| unprocessed_pseudogene | 57 0.0% | 55 0.1% | 86 0.0% | 55 0.1% | 26 0.0% | 31 0.1% |
| vaultRNA | 0 0.0% | 33 0.0% | 133 0.1% | 32 0.0% | 0 0.0% | 0 0.0% |
| mature_miRNA (%) | 87.9% | 85.4% | 87.8% | 74.5% | 89.3% | 29.1% |
