# Supplementary material for: Low-cell-number, single-tube amplification (STA) of total RNA revealed transcriptome changes from pluripotency to endothelium
Source: BMC Biol. 2017 Mar 21;15:22. doi: 10.1186/s12915-017-0359-5 (PMC5360049; doi:10.1186/s12915-017-0359-5)
Supplement: Additional file 3: — Table S2. The locations and identities of the top 40 abundant reads belonging to the unannotated category after feature assignment. The nontranscript, non-tRNA (GENCODE v22), and nonrepeat (RepeatMasker) reads of TP100N, CP100N, and CP100W were pooled together and merged into genome coordinates with the “bedtools merge” function. The numbers of reads on each coordinate were counted. The percentages (%) were defined as the counts on each coordinate divided by the summed counts of all coordinates. The features were searched by the order of human mRNA, Expressed Sequence Tag (EST), and Basic Local Alignment Search Tool (BLAST) against the human transcriptome. (DOCX 13 kb) [file 12915_2017_359_MOESM3_ESM.docx]

| Chromosome | Start | End |  | Strand | Counts | % |  | Features | Corresponding Figures |
| --- | --- | --- | --- | --- | --- | --- | --- | --- | --- |
| KI270733.1 | 123666 | 179772 | + | | 675831 | 43.0% | RNA45S5 |  |  |
| GL000220.1 | 106427 | 159518 | + | | 496260 | 31.5% | RNA45S5 |  |  |
| GL000220.1 | 159528 | 161802 | + | | 75066 | 4.8% | RNA45S5 |  |  |
| chr13 | 114181084 | 114181107 | - |  | 68490 | 4.4% | EST DB085301, intron | | Fig. S2A1 |
| KI270733.1 | 133119 | 133139 | - |  | 23221 | 1.5% | antisense, RNA45S5 | |  |
| KI270733.1 | 127678 | 127704 | - |  | 19626 | 1.2% | antisense, 18S ribosomal RNA gene | |  |
| chrM | 14684 | 14705 | + | | 17885 | 1.1% | mitochondiral reads |  |  |
| chrM | 14633 | 14674 | + | | 17098 | 1.1% | mitochondiral reads |  |  |
| chrM | 15976 | 16000 | + | | 11580 | 0.7% | mitochondiral reads |  |  |
| chr5 | 100046424 | 100046448 | + | | 9995 | 0.6% | mRNA FZ426195 |  | Fig. S2A2 |
| chr1 | 630690 | 630823 | + | | 7831 | 0.5% | antisense, RP5-857K21.4, intron | | Fig. S2A6 |
| chr21 | 8256934 | 8257030 | + | | 7579 | 0.5% | RNA28S5, intron |  | Fig. S2A3 |
| GL000220.1 | 158525 | 158549 | - |  | 7298 | 0.5% | antisense, RNA45S5 | |  |
| GL000220.1 | 118375 | 118399 | - |  | 6505 | 0.4% | antisense, RNA45S5 | |  |
| KI270733.1 | 131409 | 131433 | - |  | 6390 | 0.4% | antisense, RNA45S5 | |  |
| KI270733.1 | 178483 | 178521 | - |  | 5360 | 0.3% | antisense, EST BE813278 | |  |
| GL000218.1 | 42021 | 42047 | + | | 4830 | 0.3% | antisense, mRNA AK092792 | |  |
| chr21 | 8439976 | 8440084 | + | | 4822 | 0.3% | RNA45S5, exon |  |  |
| GL000220.1 | 159632 | 159653 | - |  | 4473 | 0.3% | antisense, mRNA BC050745 | |  |
| KI270733.1 | 130326 | 130371 | - |  | 3210 | 0.2% | antisense, mRNA AK057572 | |  |
| chr11 | 130427800 | 130427827 | + | | 2958 | 0.2% | antisense, ADAMTS8, exon | |  |
| chr6 | 12012110 | 12012137 | - |  | 2498 | 0.2% | antisense, EST BP285844, intron | |  |
| chr11 | 10509204 | 10509302 | - |  | 2489 | 0.2% | piR-32376 |  | Fig. S2A4 |
| GL000220.1 | 118134 | 118158 | - |  | 2454 | 0.2% | antisense, mRNA BC043008 | |  |
| chr10 | 8055911 | 8055934 | - |  | 2436 | 0.2% | antisense, GATA3, intron | |  |
| GL000220.1 | 113480 | 113514 | - |  | 2245 | 0.1% | antisense, mRNA BC043008 | |  |
| GL000220.1 | 110325 | 110344 | - |  | 1914 | 0.1% | antisense, mRNA BC043008 | |  |
| chr3 | 49664451 | 49664474 | - |  | 1865 | 0.1% | antisense, BSN, exon | |  |
| GL000220.1 | 117871 | 117912 | - |  | 1618 | 0.1% | antisense, mRNA BC043008 | |  |
| GL000220.1 | 118279 | 118311 | - |  | 1547 | 0.1% | antisense, mRNA BC043008 | |  |
| chrM | 5818 | 5848 | + | | 1542 | 0.1% | mitochondiral reads |  |  |
| KI270733.1 | 176407 | 176432 | - |  | 1426 | 0.1% | antisense, mRNA HV234042 | |  |
| KI270733.1 | 126367 | 126406 | - |  | 1413 | 0.1% | antisense, mRNA AK057572 | |  |
| KI270733.1 | 175581 | 175608 | - |  | 1348 | 0.1% | antisense, mRNA DQ598445 | |  |
| chrM | 5584 | 5622 | + | | 1341 | 0.1% | mitochondiral reads |  |  |
| chr21 | 8257107 | 8257332 | + | | 1295 | 0.1% | RNA28S5, intron |  |  |
| KI270713.1 | 31627 | 31709 | - |  | 1253 | 0.1% | mRNA AK125737 |  |  |
| KI270733.1 | 127178 | 127197 | - |  | 1241 | 0.1% | antisense, mRNA AK057572 | |  |
| KI270733.1 | 134734 | 134775 | - |  | 1238 | 0.1% | antisense, mRNA AK057572 | |  |
| chr1 | 632706 | 632756 | + | | 1161 | 0.1% | near the start of tRNASer(UNC) | | Fig. S2A5 |
