# Supplementary material for: Low-cell-number, single-tube amplification (STA) of total RNA revealed transcriptome changes from pluripotency to endothelium
Source: BMC Biol. 2017 Mar 21;15:22. doi: 10.1186/s12915-017-0359-5 (PMC5360049; doi:10.1186/s12915-017-0359-5)
Supplement: Additional file 7: — Table S3. Primers used for library preparation and PCR. Matched or complementary sequences are either color-coded or underlined to aid identification. (DOCX 23 kb) [file 12915_2017_359_MOESM7_ESM.docx]

| Name | Sequence | Function |
| --- | --- | --- |
| TSO | AAGCAGTGGTATCAACGCAGAGTACGCrGrG+G | attach 5' adapter to RNA |
| i7T20V | GCTCGGAGATGTGTATAAGAGACAGTTTTTTTTTTTTTTTTTTTTV | attach 3' adapter to RNA |
| PCRIIA | AAGCAGTGGTATCAACGCAGAGT | preamplify cDNA |
| FC-121-1030 | GTCTCGTGGGCTCGGAGATGTGTATAAGAGACAG |  |
| Looped oligo rA5 (LOrA5) | CCGGCGCATACGACGATTCTGTGATCTCGAGATCACAGAATCGTCGTATGCGAAAArArArArArA | test for polyadenylation |
| Looped oligo (LO) | TTCTCTCTCCCTTCCTCTGGAGAGGAAGGGAGAGAGAA | test for template switch activity |
| P5PCRIIA | AATGATACGGCGACCACCGAGATCTAAGCAGTGGTATCAACGCAGAGT | add P5 adapter sequence to libraries |
| N7xx | CAAGCAGAAGACGGCATACGAGATxxxxxxxxGTCTCGTGGGCTCGG | add P7 adapter sequence to libraries |
| Sequencing primer | AAGCAGTGGTATCAACGCAGAGTACGCGGG | sequence libraries |
| mT7A20 | CTCTCTAATACGACTCACTATAGGGAAAAAAAAAAAAAAAAAAAA | test for the limit of sensitivity of STA |
| mT7 F | CGCAGAGTACGCGGGCTCTC | amplify mT7A20 |
| mT7 R | CCCTATAGTGAGTCGTATTA |  |
| RNA27 | UUCUUCACAAGUGGUGUAUCAACGUUU | prepare mock libraries |
| RNA27 primer F | CGGGTTCTTCACAAGTGG | amplify RNA27 |
| RNA27 primer R | TTTTTTTTTTTTTTTTAAACGTTGATACAC |  |
| RNA21 | UUGUACUACACAAAAGUACUG | prepare mock libraries |
| SMART27 | AAGCAGTGGTATCAACGCAGAGTACGCGGGTTCTTCA |  |
| SMART21 | AAGCAGTGGTATCAACGCAGAGTACGCGGGTTGTAC |  |
| R2T20V | CGGTCTCGGCATTCCTGCTGAACCGCTCTTCCGATCTTTTTTTTTTTTTTTTTTTTV |  |
| R2 | GTCTCGGCATTCCTGCTGAACCGCTCTTCCGATC |  |
| miR-19B-3p F | GAGTACGCGGGTGTGCAAAT | amplify transcripts |
| miR-19B-3p R | TTTTTTTTTTTTTTTTCAGTTTTGCATG |  |
| miR-367-3p F | GAGTACGCGGGAATTGCACTTT |  |
| miR-367-3p R | TTTTTTTTTTTTTTTTTCACCATTGCTAAAG |  |
| miR-302C-3p F | GAGTACGCGGGTAAGTGCTTC |  |
| miR-302C-3p R | TTTTTTTTTTTTTTTCCACTGAAACATGG |  |
| miR-196B-5p F | CAGAGTACGCGGGTAGGTAGTTT |  |
| miR-196B-5p R | TTTTTTTTTTTTTTTCCCAACAACAGG |  |
| miR-LET7A-5p F | GAGTACGCGGGTGAGGTAG |  |
| miR-LET7A-5p R | TTTTTTTTTTTTTTTTAACTATACAACCTACT |  |
| RNU6-1 F | CAGAGTACGCGGGGTGCTCG |  |
| RNU6-1 R | TTTTTTTTTTTTTTTTTTAAAATATGGAACGC |  |
| miR-498 F | GAGTACGCGGGTTTCAAGCC |  |
| miR-498 R | TTTTTTTTTTTTTTTGAAAAACGCCCCCT |  |
| miR-515-5p F | CAGAGTACGCGGGTTCTCCAAAA |  |
| miR-515-5p R | TTTTTTTTTTTTTTTCAGAAAGTGCTTTC |  |
| Name | Sequence | Function |
| miR-519E-5p F | GAGTACGCGGGTTCTCCAAAA | amplify transcripts |
| miR-519E-5p R | TTTTTTTTTTTTTTTGAAAGTGCTCCC |  |
| miR-92A-3p F | GAGTACGCGGGTATTGCAC |  |
| miR-92A-3p R | TTTTTTTTTTTTTTTACAGGCCGGGA |  |
| miR-24-3p F | CGCGGGTGGCTCAGTTC |  |
| miR-24-3p R | TTTTTTTTTTTTTTTCTGTTCCTGCTG |  |
| miR-126-5p F | GAGTACGCGGGCATTATTAC |  |
| miR-126-5p R | TTTTTTTTTTTTTTTCGCGTACCAAAA |  |
| miR-126-3p F | GAGTACGCGGGTCGTACCG |  |
| miR-126-3p R | TTTTTTTTTTTTTTTCGCATTATTACTCAC |  |
| miR-887-3p F | GTACGCGGGGTGAACGG |  |
| miR-887-3p R | TTTTTTTTTTTTTTTCCTCGGGATG |  |
| Novel Tx F | AGTACGCGGGGTGTCCCA |  |
| Novel Tx R | TTTTTTTTTTTTTTTGGCGACCCAG |  |
| MT_TL2 F | GAGTACGCGGGACTTTTAAAG |  |
| MT_TL2 R | TTTTTTTTTTTTTTTTACTTTTATTTGGAG |  |
